# Species richness variation in marine and terrestrial fauna across widespread, fragmented territories: assessing inherent challenges of data scarcity at local and regional scales

**DOI:** 10.1038/s41598-025-06631-4

**Published:** 2025-07-01

**Authors:** Kilian Barreiro, Laura Benestan, Charlotte Moritz, Simon Ducatez, Jean-Claude Gaertner, Jérémy Le Luyer, Cristián J. Monaco

**Affiliations:** 1 IFREMER, IRD, Institut Louis-Malardé, Univ Polynésie française, UMR SECOPOL, Vairao, Tahiti, French Polynesia; 2 IFREMER, Univ Brest, CNRS, IRD, UMR-6539 LEMAR, Plouzané, Brittany, France; 3 CMOANA Consulting, BP 71607, Taravao Tahiti, 98719 French Polynesia; 4 IRD, IFREMER, Institut Louis-Malardé, Univ Polynésie française, UMR SECOPOL, Tahiti, French Polynesia

**Keywords:** Fauna, Global biodiversity information facility, French Polynesia, Marine, Terrestrial, Biodiversity, Biogeography, Data publication and archiving

## Abstract

The ongoing biodiversity crisis calls for a complete biodiversity inventory of marine and terrestrial ecosystems. The task is particularly challenging for fragmented island territories, where baseline biodiversity information is often difficult to procure. By centralising information from different sources (museums, research institutions, citizen scientists), ‘big-data’ platforms provide an opportunity to evaluate species biodiversity information of understudied regions. Using data primarily sourced from the Global Biodiversity Information Facility (GBIF), and complemented by a review of 56 potential data sources—of which nine provided unique, non-redundant records—we curated the first biogeographic dataset for both marine and terrestrial animal species in French Polynesia, a large territory composed of 124 islands and atolls that belongs to the Central Pacific region, a marine biodiversity hotspot facing conservation challenges. The dataset revealed heterogeneous species richness across archipelagos and islands, prompting an investigation into potential sampling biases (institutional, taxonomic, spatial) as well as an assessment of island-specific accessibility biases. We estimated that the archipelagos and islands had an inventory completeness rate that ranges from 1.9 to 98.4%, suggesting that a large proportion of the studied area remains poorly documented. Spatial and temporal sampling biases were partly explained by accessibility constraints (proximity to airports, roads or ports), and inventory completeness was higher for marine than terrestrial species. The biases quantified here challenge our ability to conduct biogeographic analyses that integrate the land-sea meta-ecosystem. Our database allows identifying taxa and sampling locations that require urgent attention, as well as comprehensively recorded species that can serve as indicators for environmental degradation. Explicitly acknowledging the inherent biases of biodiversity datasets is the first step towards a more comprehensive characterization of species diversity across fragmented territories. This information is crucial for guiding sound adaptive-management and conservation planning strategies.

**Supplementary Information:**

The online version contains supplementary material available at 10.1038/s41598-025-06631-4.

## Introduction

Humans are driving an unprecedented erosion of marine and terrestrial biodiversity, fundamentally altering the structure and functioning of ecosystems, and in return threatening the beneficial contributions that nature provides^1–3^. Implementing conservation actions to confront this crisis requires comprehensive and spatially explicit baseline information on species diversity across the planet^4^. Ultimately, these data are essential for guiding conservation management based on a sound understanding of the ecological and evolutionary processes that drive spatial and temporal patterns of species distribution across ecosystems^5,6^.

Thanks to the concerted efforts from museums, research institutions, citizen scientists, and ‘big-data’ platforms facilitating the integration of information, biodiversity records are increasingly available^7–9^. Over the last two decades, many initiatives to centralise species occurrence data have emerged, notably some online repositories including the Global Biodiversity Information Facility (GBIF, https://www.gbif.org/) and the Ocean Biodiversity Information System (OBIS, https://obis.org/). By adhering to the FAIR principles (Findability, Accessibility, Interoperability, and Reusability) and the metadata-sharing standards, such as the Darwin Core (DwC)^10^, Ecological Metadata Language (EML)^11^, and BioCASe^12^, these intergovernmental research infrastructures promise to expedite the study of biodiversity across ecosystems. GBIF and OBIS are the largest open-access occurrence data portals for terrestrial and marine species, both being routinely used to inform resource management and conservation programs (e.g^13–17^).

Despite their growing popularity, open-access biodiversity databases have been criticised on the grounds of poor data quality, potentially limiting their scope and applicability^18^. Important shortfalls that are often cited include standardisation issues during sampling^19,20^, incomplete and/or incorrect records (e.g., species misidentification) and sampling biases, either spatial/temporal (i.e., unbalanced sampling efforts across space/time), taxonomic (i.e., skewed sampling favouring certain taxa), or both^20–23^. While cleaning and filtering methods allow readily correcting for incomplete and/or incorrect entries, sampling biases are difficult to diagnose and require special attention^24^. The spatial sampling bias, considered one of the main challenges limiting our comprehensive understanding of large-scale biodiversity patterns^25^, can be partly explained by socio-economic reasons (e.g., wealthy zones are more likely to be surveyed^26^), a scientific bias towards certain taxa^21, ^differences in sampling standards^27^, and/or by logistical difficulties to access certain locations^28,29^. Notably, implementing standardized sampling methods in future research is essential, as it enhances comparability and integration, increases the reproducibility of findings, and improves data quality and reliability, while also saving time and resources^30^. These shared data collection protocols should be adopted and facilitated for both scientific and non-scientific personnel^31^.

Remote oceanic islands are likely to show sampling gaps due to their geographical isolation, which ultimately results in patchy and poorly representative data for the study region. The difficulties and high costs associated with organising monitoring campaigns further exacerbate these biases. As a result, some islands are underrepresented in long-term monitoring schemes^32^ and, aside from a few exceptions (e.g.,^33^), comprehensive biodiversity studies across widespread archipelagos remain rare. This paucity of information for islands and atolls is particularly detrimental because they are a priori highly vulnerable ecosystems that potentially harbour high levels of endemism due to their isolation^34,35^. Additionally, fragmented archipelagos are unique natural laboratories that provide opportunities for studying the ecological and evolutionary processes driving biodiversity patterns, dispersal potential, endemism and extinction rates, for both marine and terrestrial organisms. However, a proper understanding of these biogeographical processes first requires robust baseline information on species distribution^36,37^.

With 124 high islands and atolls spread across five archipelagos covering 4.8 million km^2^^38,39^, French Polynesia represents the epitome of a fragmented territory. The large number of islands, their relative isolation, and the sheer variation in geomorphological characteristics they exhibit complicate efforts to survey the entire region or avoid sampling biases. Indeed, the marine and terrestrial biogeography of French Polynesia has only been partly studied, with a remarkable skew towards specific taxonomic groups. In the marine realm, targeted investigations have mainly focused on marine molluscs, brown seaweeds (*Phaeophyceae*), and reef fishes^40–48^. In the terrestrial realm, data compilations include a checklist of the recorded land and fresh-water arthropods^49^, a biogeographic atlas of birds^50^, and an inventory of the vascular flora^51–53^, as well as some rare studies focusing on the phylogeographic origins of specific terrestrial biota (e.g.,^54,55^). Overall, the lack of a centralised, complete, and unbiased dataset for the region prevents an exhaustive analysis of the biogeographical status of marine and terrestrial species across French Polynesia. As a model of a highly fragmented island system, improving our fundamental understanding of French Polynesian biogeography is not only critical for cataloguing the existing fauna of the region, but also for contributing to our general comprehension of the ecological processes driving the current biodiversity crisis in isolated systems^35,56^.

Using data originally downloaded from open-access portals (GBIF, OBIS) and 56 additional occurrence data sources, we compiled and curated the first biogeographic dataset for both marine and terrestrial animal species in French Polynesia. We used these data to: (1) provide a baseline characterization of the number of species in the region; (2) identify taxonomic groups that might require further investigation, as well as comprehensively recorded species that can serve as indicators for environmental degradation; (3) identify poorly- and well-surveyed islands; and (4) quantify island-specific accessibility biases leading to heterogeneous sampling efforts.

## Materials and methods

### Data collection

**Fig. 1:**
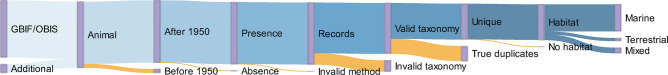
Sankey diagram illustrating the data filtering and quality-control steps. To obtain the final marine, terrestrial and mixed-habitat animal dataset, we downloaded data from GBIF/OBIS data portals and from additional occurrence data sources (Table S2), we removed: records earlier than 1950 (20,967 occurrences), absence data (< 1000 occurrences), occurrences based on invalid recording methods (< 1000 occurrences), invalid taxonomical information (58,490 occurrences), and true duplicates (77,651 occurrences). Finally, data with unavailable habitats were excluded from subsequent analyses (< 1000 occurrences). The cleaned dataset is available in SEANOE (https://www.seanoe.org/data/00878/99018/).

We downloaded occurrence data from the GBIF portal (http//gbif.org; 10.15468/dl.gaxgr7) on May 24, 2023, covering French Polynesia (polygon spanning between 5°S and 30°S, and 134°W and 155°W). To identify any additional biodiversity records not included in GBIF, we reviewed 56 data sources, including local reference guides, expedition reports, and repositories (Table S2). Nine sources contained unique georeferenced species records that were integrated into our final dataset. The remaining sources were used to cross-check metadata, validate completeness, or support contextual interpretation. Species occurrences are defined as records of a particular species (or other taxonomic rank), with a geographic location and timestamp. These raw data were treated following the Darwin Core^10^, Ecological Metadata Language^11^, and BioCASe^12^ standards. A pre-filtration of the data was done to exclude records missing geographic location and/or taxonomic classification (e.g., not available or zeros), yielding 343,780 records (Fig. 1). Because GBIF and OBIS signed a data-sharing agreement which was effective at the time we downloaded the data, the marine data from OBIS was also contained in our GBIF data. The coastline shapefiles used to analyse the region included 120 geographical structures, most of which were atolls and high islands. Hereafter, we refer to all geographical structures as “islands”. Each record retrieved from the GBIF dataset was assigned to its nearest island based on geographic distances estimated using the function *st_nearest_feature* available in the *sf* package v.1.0-15^57^ in R^58^.

### Validation of the taxonomic information

To clean, homogenise, and validate the taxonomic information in the dataset, we assumed that misidentifications would occur at the species level. To ensure taxonomic reliability we validated each species name using ad hoc taxonomic data repositories. We first validated the species name of each recognized taxon with WoRMS (*World Register of Marine Species*, https://www.marinespecies.org/) using the *wm_records_name* function from the R package *worrms*^59^. We then assigned a taxonomic status (i.e., “accepted”, “doubtful”, “synonym”) to each record following the criteria outlined by the GBIF Backbone taxonomy (see 10.15468/39omei, https://hosted-datasets.gbif.org/datasets/backbone/). Taxa that were assigned as either “doubtful” or “synonym” were replaced by the updated taxonomic name provided by WoRMS. Taxa not recognized by the WoRMS repository were further examined using the *gna_verifier* function from the R package *taxize*^60^, which provides a means to validate species names by accessing several additional repositories via specific Application Programming Interfaces (e.g., ITIS: *Integrated Taxonomic Information System*, https://www.itis.gov/; *CoL*: Catalogue of Life, https://www.catalogueoflife.org/; bold: *Barcode of Living Data*, https://www.boldsystems.org/). Taxa that were not recognized neither by WoRMS nor Taxize were submitted to TAXREF (taxonomic reference curated by the French National Museum of Natural History, https://inpn.mnhn.fr/programme/referentiel-taxonomique-taxref) using the *rt_taxa_search* function from the *rtaxref* R package^61^. A final manual check was done for records that could not be identified in the aforementioned taxonomic repositories.

### Habitat classification and biogeographical status

Habitat classification for marine and terrestrial species were verified using WoRMS and Taxref, respectively. Habitat information was split into four categories (i.e., marine, brackish, freshwater and terrestrial) according to the classification scheme favoured by WoRMS. Missing habitat information was completed using the TaxRef database. For our analyses of terrestrial and marine ecosystems, we focused on species that were classified as exclusively “marine” or exclusively “terrestrial”. Species classified as amphibious or those inhabiting both terrestrial and marine environments at different life stages (e.g., seabirds like *Gygis alba* or *Sula sula*) or during specific phases of their life cycle (e.g., insects with aquatic larval stages) were included in the cleaned dataset (labeled as “Mixed” in Fig. 1) but excluded from further analyses.

### Data filtration sequence

Because geographic, taxonomic and accuracy standards have changed over time^20,62^, and notorious errors were detected in older records, we retained entries dating from 1950 onwards and excluded those without timestamps (Fig. 1). Subsequently, we removed all absence data to rule out potential biases due to false negatives and no-observation data^63^. We then restricted occurrences to those described in the *basisOfRecords* column as: “human observation”, “machine observation”, “material sample”, “material citation” and “preserved specimen” according to recommendations by Smith et al. 2018^64^. Cross-checking values corrected real duplicates in *decimalLatitude*,* decimalLongitude*,* ScientificName*,* Year*,* Month* and *Day* categories. Finally, records lacking *species* names and *Habitat* information were removed from the dataset (Fig. 1).

### Taxonomic biases: identifying under- and over-represented groups

We estimated the taxonomic bias at the *Class* level based on its over- or under-representation, relative to an “ideal sampling effort index”. The ideal number of records for a given class was estimated based on the hypothetical scenario where each species received the same number of records, and therefore each class received a number of records directly proportional to its number of species^21^ according to:

Ideal = N_rec_ * (N_sp_group_/ N_sp_tot_)

where N_rec_ = total number of records, N_sp_group_ = number of distinct species within the taxonomic group, and N_sp_tot_ = total number of species present in the whole dataset. Taxonomic bias was assessed based on the difference between the ideal and observed sampling efforts, calculated for each class with more than 100 records in marine habitats and more than ten records in terrestrial habitats. To highlight values that deviated significantly from the ideal, we applied an inverse hyperbolic sine transformation to the data. We also identified the top ten most representative species for each habitat.

### Spatial and temporal heterogeneity in the sampling effort

To examine spatial heterogeneity in the sampling effort, we first mapped the number of records and species estimated for each island within each archipelago. The agreement between the number of records and species per island (log_10_-transformed) was evaluated based on the Pearson correlation coefficients and its statistical significance. Additionally, to quantify the prevalence of heterogeneous sampling effort across space, we assessed the proportion of species recorded only once at each island, a common method in biodiversity studies to evaluate sampling completeness and detect potential under-sampling biases^65^. The parameter uniqueness—species that have only been collected once—is widely recognized as an indicator of incomplete sampling^66,67^, allowing researchers to infer the adequacy of the sampling effort and identify areas that may require further investigation. We considered q_k_ as the number of species documented in *k* sampling-effort units, so that the number of species observed in a single sampling-effort unit is q_1_ (i.e., unique), the number of duplicates is q_2_, and so on.

### Inventory completeness

To investigate the degree of inventory completeness in the dataset for both marine and terrestrial ecosystems at the scale of the archipelago and the island, we estimated the species inventory completeness percentage (*C*), calculated as:

*C*_(*i*)_ = (*Sobs*_(*i*)_/ *Sest*_(*i*)_) * 100

where i = each island or archipelago, *Sobs* = number of species observed, and *Sest* = number of species estimated at each archipelago and island^68^. To estimate *Sest*, we used the Species Accumulation Curve (SAC) approach that describes the relationship between species richness and sampling effort, i.e., the number of records available in a grid cell^69^. To derive the SACs, we split the area in 0.05° (~ 25 km^2^) grid cells. We described the SACs using the *specaccuml* function (method = “exact”) available in the R package *vegan* v.2.6–4^70^. We fitted the Michaelis-Menten model with the *fitspecaccum* function (method = “michaelis-menten”) to provide estimates of the number of species likely to be present (i.e., *Sest*, which corresponds to the asymptotic richness, or parameter *Vm* in the Michaelis-Menten equation) and the number of records required to capture 50% (*K*) of the estimated number of species predicted by the model^71–73^. Using the *poolspecaccum* function available in the vegan package, we also compared this expected number of species with the nonparametric richness estimators, Chao1 and Jackknife 2 estimators that are recommended when the data contain a high number of unseen species^74^. Because biodiversity assessments can be biased by grid cells with extremely low species records, we considered a minimum threshold of ten observations to run the SACs, as was done in previous studies^68,75^.

While we aimed to calculate the SACs for each island and archipelago based on grid cells, as recently done in several studies on macroecology using GBIF datasets^75–77^, only eight islands (i.e., Anaa, Huahine, Moorea, Nuku Hiva, Raiatea-Tahaa, Rangiroa, Tahiti and Ua Pou) were sufficiently large to yield enough cells (greater or equal to 10) to fit the Michaelis-Menten model for terrestrial data. We therefore generated archipelago-scale models based on 5-km grid cells, while for the island-scale models we used the geographic coordinates associated with the species records. A preliminary comparison between these two approaches (0.05-degree resolution grid cells vs. records) revealed a significant correlation between them for the archipelago scale (*R*^2^ = 0.96). Therefore, we only presented SACs based on records for both archipelagos and islands. To evaluate inventory completeness, we determined the total number of islands with more than 100 records and *C* greater or equal to 80%, meaning that at least 80% of the species have been sampled^77,78^. We then examined the correlation between the number of records and *C* to test whether these proxies of sampling effort and reliability were associated. We used a Spearman correlation test for non-parametric data. Statistical significance was evaluated based on ⍺ = 0.05.

### Sampling bias due to accessibility

To explore the influence of accessibility constraints on these sampling biases, we used a Bayesian approach to estimate how sampling rates vary with proximity to several common anthropic accessibility factors (i.e., rivers, roads, cities, airports, and ports). Using the *calculate_bias* function from the *sampbias* R package v. 2.0.0^79^, we estimated the bias weights (*w*), which quantify the impact of each accessibility factor on sampling rates. These weights are calculated assuming an exponential decline in sampling rates as distance from accessibility factors increases. This package also provides spatially explicit estimates of the number of records (i.e., expected records) using a Poisson sampling process while accounting for the influence of the accessibility factors. Because the geospatial data contained by default in the *sampbias* package is incomplete for French Polynesia (Natural Earth Data, https://www.naturalearthdata.com/), we manually inputted vector data for rivers, roads, cities with > 1,000 inhabitants, airports, and ports. These data were provided by the French Polynesian agency for marine resources, the *Direction des Ressources Marines*. We defined a grid (*inp_raster* parameter) contained within the same polygon used for downloading the GBIF data, with 0.05 degrees resolution (~ 5.5 km). This was done for consistency with the SAC analyses. Each grid cell was assigned to the nearest island based on geographic distances estimated using the function *st_nearest_feature* available in the *sf* R package v.1.0-15^57^.

## Results

### Curated dataset for French Polynesian marine and terrestrial species

From the original 343,780 records included in the dataset, we removed 20,967 records that were either dated before 1950, or which did not have a time stamp (Fig. 1). Then, 58,490 records with no or non-usable species taxonomy were discarded, 86.6% of which sourced from *institutions* sources (e.g., *Museum national d’Histoire naturelle*, *Smithsonian Institution*). A total of 77,651 records were identified as duplicated, 21.5% of which originated from *citizen science* sources. The number of records accessible per year has increased over time since 1950, reaching maximum values in 2011, 2012, and 2006, with 20,636, 11,263, and 11,039 records, respectively (Fig. S1). This increase in records was mainly explained by the punctual contribution of two out of 130 publishers: OBIS-SEAMAP and UMS PatriNat (OFB-CNRS-MNHN, Paris). The mean number of records per species was 25.8 (median = 3), ranging from 1 to 12,339. Records produced by citizen scientists accounted for 21.7% of the total, corresponding to 40,394 records. Data collected by citizen scientists were also the main source of data (i.e., > 50% of occurrences sourced as citizen science) for 62 islands, and the only source (100%) for two islands (Fangatau, Marutea nord). *Human observation*, including institutional and citizen science publishers, was the most frequently used recording method, with 75.7% (140,650 records) of total records. *Preserved specimen* and *material sample* categories accounted for 20.1% and 3.9% of records, respectively. WoRMS validated the taxonomy of 90.6% of total records and 99.1% of non-terrestrial records. Only 268 species lacked information on their habitat, which we completed manually. The resulting cleaned dataset was composed of 185,758 records including 141,181 marine, 15,940 terrestrial and 28,637 mixed records for 5,953 marine, 1,032 terrestrial and 203 mixed species, collected from 1950 to 2023 (Figs. 1 and S1). The curated dataset is available in SEANOE (https://www.seanoe.org/data/00878/99018/*).*

### Taxonomic composition and biases

**Fig. 2:**
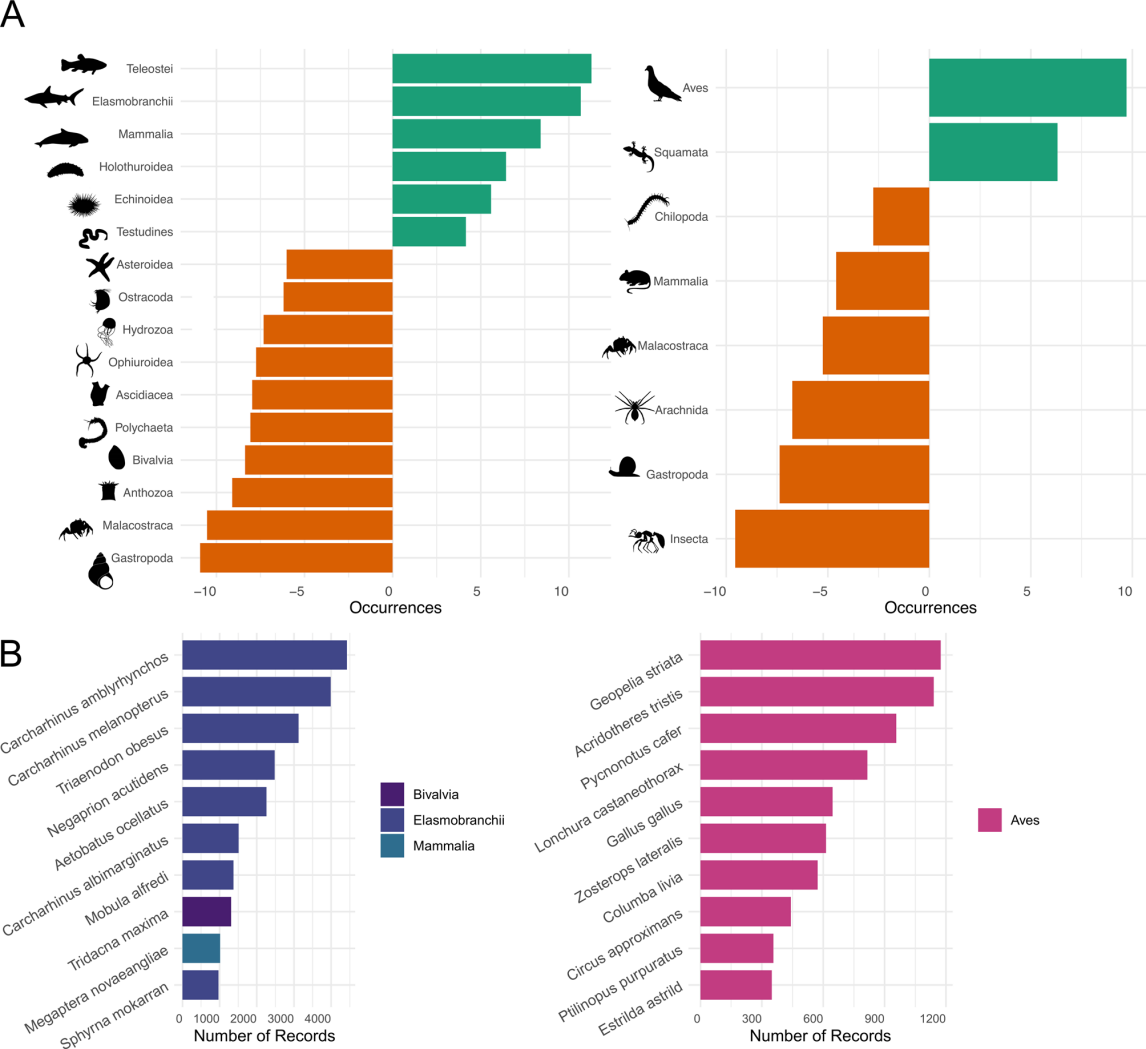
Taxonomic bias assessment. (**A**) Major class representation in sampling effort (i.e., observed - ideal). Over- and under-representation of each class are illustrated by the green and orange bars respectively. An inverse hyperbolic-sine transformation was used for the x-axis. (**B**) Number of records for the top 10 most-sampled marine (left) and terrestrial (right) species.

The number of recorded species was ~ 5.8 times higher for marine than terrestrial ecosystems, with 5,953 marine and 1,032 terrestrial species, respectively. For marine taxa, the dataset included 18 phyla, with three major groups: Mollusca (2,337 species), Chordata (1,733 species), and Arthropoda (1,148 species), accounting for over 95.4% of marine records (134,734 records). Five classes alone accounted for 78.4% of the observations: Teleostei (76,248 records, 1,547 species), Gastropoda (20,446 records, 2,028 species), Malacostraca (6,788 records, 1,076 species), Bivalvia (4,368 records, 276 species), and Mammalia (2,807 records, 25 species; Fig. 2). The most represented marine species were *Carcharhinus amblyrhynchos* (grey reef shark, 4,428 records), *Carcharhinus melanopterus* (blacktip reef shark, 3,987 records), and *Triaenodon obesus* (whitetip reef shark, 941 records; Fig. 2). A total of 90% of the marine species had 33 or fewer records, and 26% were unique records.

The terrestrial taxa comprised five phyla, including Arthropods (761 species), Mollusca (194 species), Chordata (73 species), Platyhelminthes (3 species), and Nematoda (1 species). The five most recorded classes were Aves (9,061 records, 62 species), Insecta (3,545 records, 687 species), Gastropoda (2,198 records, 194 species), Arachnida (532 records, 62 species), and Squamata (430 records, 10 species), representing 98.9% of all terrestrial species records. A total of 90% of the terrestrial species had 23 records or fewer, and 41% were unique records. Three introduced bird species, *Geopelia striata* (zebra dove), *Acridotheres tristis* (common myna), *Pycnonotus cafer* (red-vented bulbul), were the most recorded terrestrial species, with 1,174, 1,140 and 957 occurrences (Fig. 2), of which 93.1% were provided by the “Cornell Lab of Ornithology”.

### Spatial and temporal heterogeneity in sampling effort and the number of recorded species

We observed a significant and strong correlation between the log-10 number of records per island (i.e., a proxy for sampling effort) and the number of species per island for both marine (ρ = 0.984, *P* < 0.001) and terrestrial (ρ = 0.969, *P* < 0.001) ecosystems (Fig. S2). This analysis excluded islands that lacked records in both marine and terrestrial habitats.

**Fig. 3:**
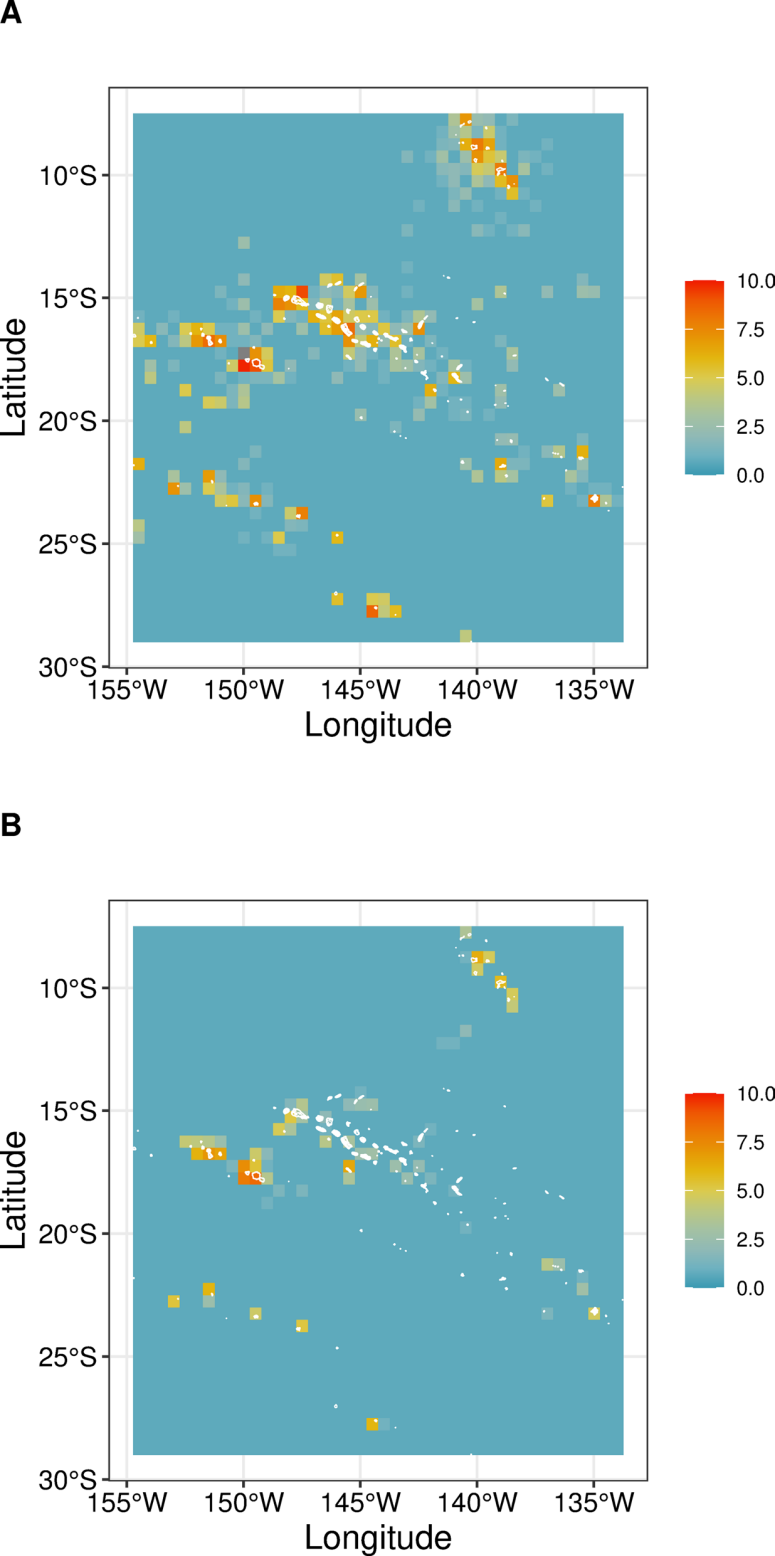
Number of database records (log-10 transformed) across French Polynesia for marine (**A**) and terrestrial (**B**) species. Blue and red colours indicate a low and high number of occurrences (proxy of sampling rate) respectively, for a 0.05-degree resolution.

**Fig. 4:**
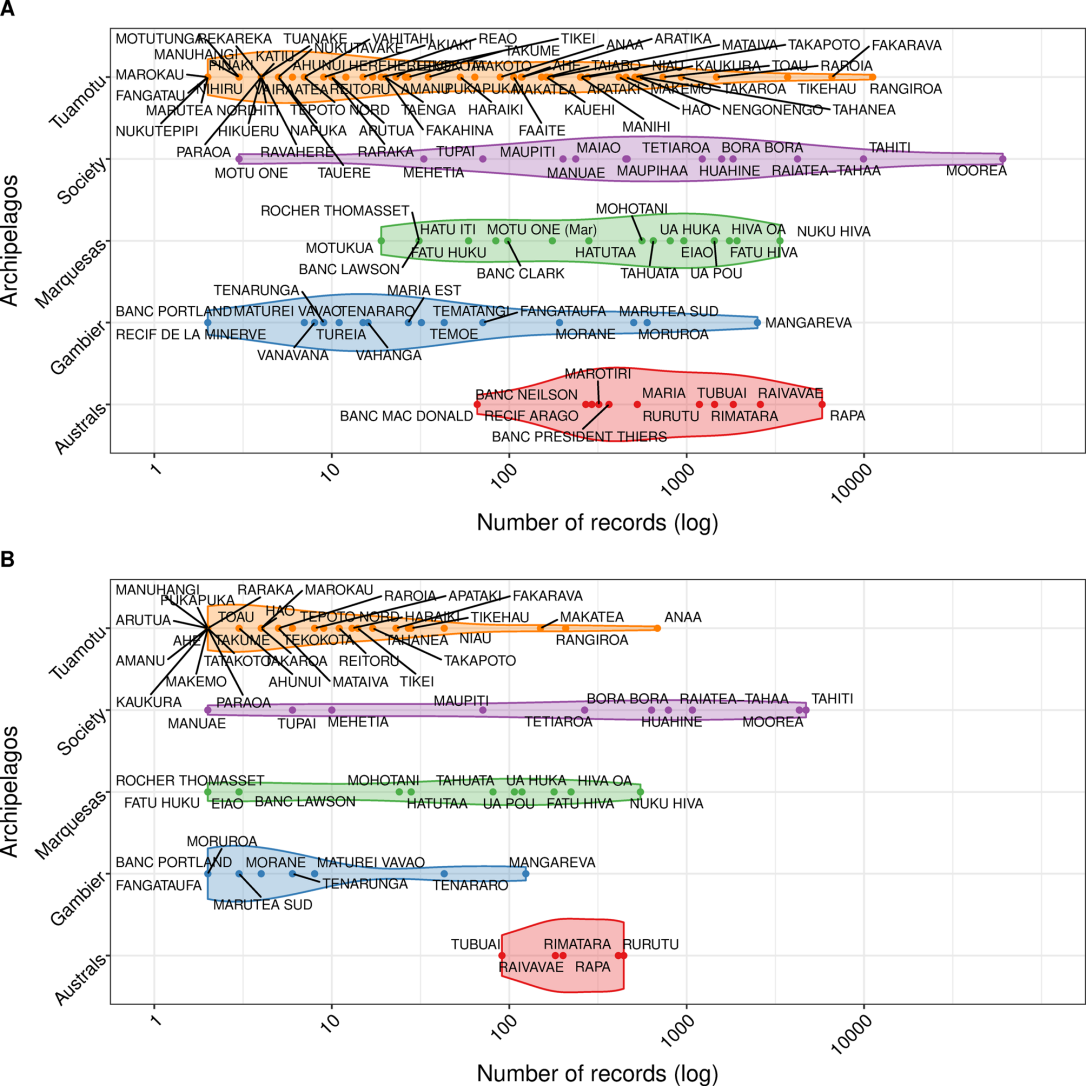
Number of marine (**A**) and terrestrial (**B**) records per island and archipelago. Islands without records are excluded. The x-axis is presented on a logarithmic scale.

Our dataset included marine species records for 118 out of 124 islands. The number of records per island was heterogeneous (Fig. 3), ranging from 1 to 60,473, with a mean of 1,196 records (median = 77). The number of species present was also highly heterogeneous across space, ranging from 1 to 2,770 species per island, with a mean of 199 species (median = 58) per island. The Society Archipelago (13 islands) held 57.1% of all marine-species records, 75.0% of which were observed in Moorea (60,473 records), Tahiti (9,920 records), and Raiatea-Tahaa (4,213 records; Fig. 4). Considering the other four archipelagos, the islands that exhibited the highest number of records were Rapa (5,797 records) in the Austral islands (11 islands), Fakarava (6,585 records) in the Tuamotu (69 islands), Nuka Hiva (3,356 records) in the Marquesas (17 islands), and Mangareva (2,504 records) in the Gambier (11 islands; Fig. 3). Gambier was the least sampled archipelago, accounting for 2.9% of all marine records, and for only 13.3% of all marine species identified.

Considering the terrestrial habitat, our dataset identified 68 islands with at least one species record, and 52 islands with no records. As for the marine habitat, the number of terrestrial species records per island was heterogeneous (Fig. 3), ranging from 1 to 4,705, with a mean of 234 records per island (median = 8.5). The number of species identified per island ranged from 1 to 384, with a mean of 34 species per island (median = 4.5). The Society Archipelago held 74.4% of all terrestrial species records, 85.2% of which were registered in the trio Moorea (4,314 records, 384 species), Tahiti (4,705 records, 301 species), and Raiatea-Tahaa (1,076 records, 201 species; Fig. 4). Considering the other four archipelagos, the islands showing the highest number of records were Anaa (683 records) in the Tuamotu, Rurutu (441 records) in the Austral, Nuku Hiva (548 records) in the Marquesas, and Mangareva (123 records) in the Gambier (Fig. 4). As for the marine database, the Gambier archipelago had the lowest number of terrestrial records, representing only 4.7% of all terrestrial species identified.

### Inventory completeness

Considering the archipelago scale, the SAC analysis showed that the number of species recorded increased with sampling effort. Although the curves for both marine and terrestrial datasets exhibited a plateau, they did not reach a clear saturation point (Fig. S3). Our calculations suggest that marine inventory completeness was comparable among archipelagos, with 76.6%, 74.9%, 75.6%, 79.9% and 76.6% for the Austral, Gambier, Marquesas, Society and Tuamotu Archipelagos, respectively, indicating that at least 70% of the species were detected overall. According to the asymptote values based on the Michaelis-Menten model (*Sest*), marine species richness was lowest at the Gambier (*Sest =* 1,055, Chao1 = 1,221, Jackknife 2 = 1,317 species) and the highest at the Society (*Sest =* 5,230, Chao1 = 6380, Jackknife 2 = 6,969 species) archipelagos. The Austral, Marquesas and Tuamotu Archipelagos showed similar asymptote values of 2,538 (Chao1 = 3,067, Jackknife 2 = 3,291), 2,648 (Chao 1 = 3,497, Jackknife 2 = 2,120), and 2,120 (Chao1 = 2,639, Jackknife 2 = 2,807) expected species, respectively (Table 1).

**Table 1:** Archipelago-scale Michaelis-Menten model output parameters based on a records approach (*N* records as sampling units).

			Records approach			
Ecosystem	Archipelago	*Sobs*	*Sest*	K	C	N
Marine	Austral	1,881	2615.52	4036.62	75.59	14,679
	Gambier	788	1023.53	1324.89	76.99	4,026
	Marquesas	1,929	2744.47	4294.65	71.42	12,196
	Society	4,160	5174.48	20207.04	80.41	80,682
	Tuamotu	1,602	2027.48	5042.25	79.01	29,598
Terrestrial	Austral	283	389.51	561.55	72.65	1,323
	Gambier	49	76.49	98.18	64.06	185
	Marquesas	275	571.69	1402.90	48.10	1,307
	Society	613	728.29	2684.62	84.04	11,866
	Tuamotu	102	120.71	301.91	84.50	1,259

Analyses are per Archipelago and for each ecosystem. Parameters provided are the number of observed species (*Sobs*), the maximum number of species estimated (*Sest*), the number of records required to capture 50% of the maximum number of species estimated (*K*), and the inventory completeness in percentage (*C*).

Inventory completeness for terrestrial species was highly heterogeneous across archipelagos, ranging from 43.0% for the Marquesas, the northernmost and most remote archipelago, to 82.1% in the Society. Inventory completeness for terrestrial species was higher than for marine species in the Society (*C* = 82.1% versus 79.9%) and Tuamotu Archipelagos (*C* = 81.5% versus 76.6%), but lower in the Gambier (*C* = 64.1% versus 74.9%) and Marquesas (*C* = 43.0% versus 75.6%). For terrestrial species, the asymptote values based on the Michaelis-Menten model (*Sest*) ranged from 76 (Gambier, Chao1 = 73, Jackknife 2 = 84) to 640 species (Marquesas, Chao 1 = 639, Jackknife 2 = 609), with 484 (Chao 1 = 595, Jackknife = 578), 606 (Chao 1 = 894, Jackknife = 981), and 100 (Chao 1 = 152, Jackknife = 165) species estimated for the Austral islands, Society and Tuamotu, respectively.

At the island scale and for the marine dataset, we fitted SACs for 73 out of 119 islands having at least 10 records (Table 2). Inventory completeness was highly heterogeneous, ranging from 1.9% (Takaroa, Tuamotus, 12 records) to 82.2% (Moorea, Society, 2,826 records), with an average (± SD) of 39.2% (± 20.0%). Assuming a threshold of *C* ≥ 80% and at least 100 records, only two islands were classified as well-sampled: Moorea (2,826 records, *C =* 82.2%) and Fakarava (520 records, *C =* 82.0%). Among the islands with the highest number of records, we identified low to moderate inventory completeness for Tahiti (1,226 records, *C =* 65.9%), Bora-Bora (521 records, 60.8%) and Raiatea-Tahaa (263 records, *C =* 28.5%) in the Society, Rapa (385 records, *C* = 75.6%) and Raivavae (352 records, *C* = 69.1%) in the Austral Islands, Nuku Hiva (376 records, *C* = 65.8%) and Hiva Oa (234 records, *C* = 68.6%) in the Marquesas, Tikehau (360 records, *C* = 62.6%) and Rangiroa (343 records, *C* = 52.0%) in the Tuamotu. The correlation between inventory completeness and the number of records per island was moderate (R² = 0.41; P-value < 0.001).

**Table 2:** Island-scale Michaelis-Menten model output parameters based on a records approach (N records as sampling units) for the marine ecosystem.

Island	Sobs	Sest	K	C	*N*
AHE	103	379.44	784.20	27.14	116
AMANU	17	27.50	155.50	61.81	23
ANAA	112	146.67	429.35	76.36	87
APATAKI	175	483.86	571.29	36.17	213
BANC CLARK	84	270.50	484.44	31.05	96
BANC LAWSON	32	53.13	139.61	60.23	30
BANC MAC DONALD	29	62.29	97.23	46.55	34
BANC NEILSON	85	162.55	161.77	52.29	148
BANC PRESIDENT THIERS	122	250.41	261.07	48.72	200
BORA BORA	635	995.59	2098.04	63.78	1657
EIAO	349	1007.21	1078.27	34.65	532
FAAITE	116	1065.12	1192.85	10.89	104
FAKAHINA	12	16.83	57.23	71.30	19
FAKARAVA	489	587.76	1075.76	83.20	3739
FANGATAUFA	53	126.73	179.44	41.82	70
FATU HIVA	1243	4601.19	6291.63	27.01	1504
FATU HUKU	73	191.57	418.02	38.11	74
HAO	207	514.68	949.77	40.22	399
HARAIKI	69	216.66	296.69	31.85	53
HATU ITI	51	145.37	178.43	35.08	58
HATUTAA	206	761.01	950.68	27.07	198
HEREHERETUE	17	72.91	76.28	23.32	13
HIVA OA	660	1244.07	1676.62	53.05	1181
HUAHINE	849	1591.33	2462.25	53.35	1381
KAUEHI	39	66.16	134.54	58.95	65
KAUKURA	169	361.51	495.58	46.75	272
MAIAO	154	335.14	781.80	45.95	292
MAKATEA	131	221.45	495.28	59.15	95
MAKEMO	199	332.88	421.34	59.78	442
MANGAREVA	734	1048.09	1410.89	70.03	2490
MANIHI	142	328.86	455.88	43.18	223
MANUAE	149	365.42	341.95	40.77	237
MARIA	219	395.93	407.31	55.31	491
MARIA EST	29	91.69	192.61	31.63	26
MAROTIRI	109	209.67	230.64	51.99	165
MARUTEA SUD	180	260.99	303.74	68.97	502
MATAIVA	107	282.83	442.22	37.83	132
MAUPIHAA	264	575.39	776.64	45.88	430
MAUPITI	157	400.49	612.72	39.20	129
MEHETIA	45	64.77	132.15	69.47	33
MOHOTANI	242	385.00	494.80	62.86	560
MOOREA	2759	3279.32	11526.00	84.13	54,761
MORANE	168	451.15	595.76	37.24	190
MORUROA	370	890.29	945.25	41.56	598
MOTU ONE (Mar)	135	572.28	713.19	23.59	140
MOTUKUA	19	32.05	33.94	59.28	18
NENGONENGO	176	243.46	269.42	72.29	537
NIAU	155	312.98	460.46	49.52	180
NUKU HIVA	1103	1780.24	2657.89	61.96	2741
PUKAPUKA	70	270.77	253.29	25.85	64
RAIATEA-TAHAA	2598	7575.24	11475.92	34.30	3829
RAIVAVAE	634	1013.92	2085.85	62.53	2483
RANGIROA	898	1608.88	2861.20	55.82	2166
RAPA	1027	1514.15	2297.56	67.83	3731
RAROIA	417	592.38	703.65	70.39	1406
REAO	17	37.44	60.20	45.41	14
RECIF ARAGO	103	238.31	379.73	43.22	157
REITORU	45	103.56	307.88	43.45	17
RIMATARA	1158	3642.02	4230.51	31.80	1664
ROCHER THOMASSET	32	61.25	299.27	52.24	21
RURUTU	543	943.70	1341.85	57.54	905
TAHANEA	218	304.68	327.19	71.55	530
TAHITI	1337	1926.10	3205.79	69.40	6722
TAHUATA	410	971.02	1363.12	42.22	554
TAIARO	83	151.51	254.60	54.78	162
TAKAPOTO	176	393.65	474.64	44.71	252
TAKAROA	246	312.78	310.40	78.65	938
TATAKOTO	37	90.91	217.39	40.70	34
TEKOKOTA	45	175.27	362.37	25.67	22
TEMATANGI	40	93.96	164.33	42.57	42
TEMOE	37	90.72	148.68	40.79	27
TENARARO	41	47.76	85.83	85.85	17
TEPOTO NORD	36	50.37	58.03	71.47	21
TETIAROA	445	607.43	1223.13	73.26	1253
TIKEHAU	637	898.12	1529.56	70.93	3053
TIKEI	42	131.02	454.24	32.06	16
TOAU	222	321.87	347.30	68.97	623
TUBUAI	500	792.99	1222.56	63.05	1230
TUPAI	83	271.05	828.04	30.62	73
TUREIA	14	20.29	43.38	69.00	10
UA HUKA	503	1052.07	1509.22	47.81	851
UA POU	613	1213.43	1925.82	50.52	1078
VAHANGA	19	42.51	124.54	44.70	15

Parameters provided are the number of observed species (*Sobs*), the maximum number of species estimated (*Sest*), the number of records required to capture 50% of the maximum number of species estimated (*K*), and the inventory completeness in percentage (*C*).

For the terrestrial dataset, 27 islands had sufficient records (> 10 records) to fit SACs (Table 3). Inventory completeness ranged from 27.3% for the Fakarava Atoll (12 records, Tuamotu) to 98.4% for Anaa Atoll (67 records, Tuamotu). Other well-sampled islands (*C* ≥ 80% and 1 records) included Ua Huka (39 records, *C* = 87.5%), Hatutaa (23 records, C = 88.7%) and Tahuata (25 records, C = 88.4%) in the Marquesas, Tenararo (26 records, C = 97.5%) in the Tuamotu. Islands with the highest number of records, including Moorea (851 records, *C =* 76.8%) and Tahiti (1,039 records, *C* = 68.9%, respectively) were nearly well-sampled. Terrestrial species inventory completeness and sampling effort were not correlated across these islands (R² = 0.20; P-value > 0.05).

**Table 3:** Island-scale Michaelis-Menten model output parameters based on a records approach (N records as sampling units) for the terrestrial ecosystem.

Island	Sobs	Sest	K	C	*N*
ANAA	29	29.78	26.48	97.39	694
UA HUKA	15	16.74	18.12	89.59	114
TENARARO	5	5.61	10.13	89.09	42
HATUTAA	4	4.55	5.79	87.84	26
TAHUATA	13	15.02	12.98	86.53	79
RAIVAVAE	32	37.81	44.44	84.64	201
RIMATARA	50	62.47	59.03	80.04	225
MOHOTANI	6	7.69	7.01	78.06	23
TIKEHAU	7	9.15	9.87	76.50	26
RURUTU	113	151.07	174.10	74.80	470
TAKAPOTO	5	6.69	5.77	74.70	16
BORA BORA	54	74.67	275.37	72.32	628
MOOREA	527	746.89	1907.54	70.56	4341
MAKATEA	39	57.69	75.58	67.60	151
UA POU	30	46.17	62.60	64.97	102
TAHITI	393	621.56	2899.46	63.23	4699
TETIAROA	72	115.44	170.00	62.37	266
RANGIROA	62	99.61	131.25	62.24	211
TUBUAI	37	59.94	65.72	61.73	100
RAIATEA-TAHAA	268	441.26	728.54	60.74	1070
MANGAREVA	47	80.13	79.56	58.65	113
HUAHINE	239	409.14	579.88	58.42	786
NIAU	17	31.32	37.64	54.28	42
TAHANEA	9	17.19	23.96	52.35	24
RAPA	182	363.61	406.82	50.05	402
HARAIKI	8	18.29	16.43	43.74	13
NUKU HIVA	133	322.48	779.53	41.24	527
FATU HIVA	90	255.38	326.85	35.24	175
MAUPITI	45	133.07	138.71	33.82	70
HIVA OA	118	362.32	469.02	32.57	222
FAKARAVA	17	67.82	69.42	25.07	23
TIKEI	12	140.60	139.25	8.54	13

Parameters provided are the number of observed species (*Sobs*), the maximum number of species estimated (*Vm*), the number of records required to capture 50% of the maximum number of species estimated (*K*), and the inventory completeness in percentage (*C*).

Some islands exhibited contrasting patterns between terrestrial and marine inventories. For instance, Anaa was well sampled for terrestrial species (67 records, *C* = 98.4%) but only moderately sampled for marine species (31 records, *C* = 17.6%). Fakarava showed the opposite trend, with an almost complete marine inventory (*C =* 83.20%), while its terrestrial inventory was sparse (12 records, *C =* 27.3%). Overall, our results indicate that the observed species richness exceeds the Michaelis-Menten estimate by a factor of one to three, suggesting that even our current estimates likely underestimate the true species diversity. This reinforces the notion that species inventories in this region remain incomplete.

**Fig. 5:**
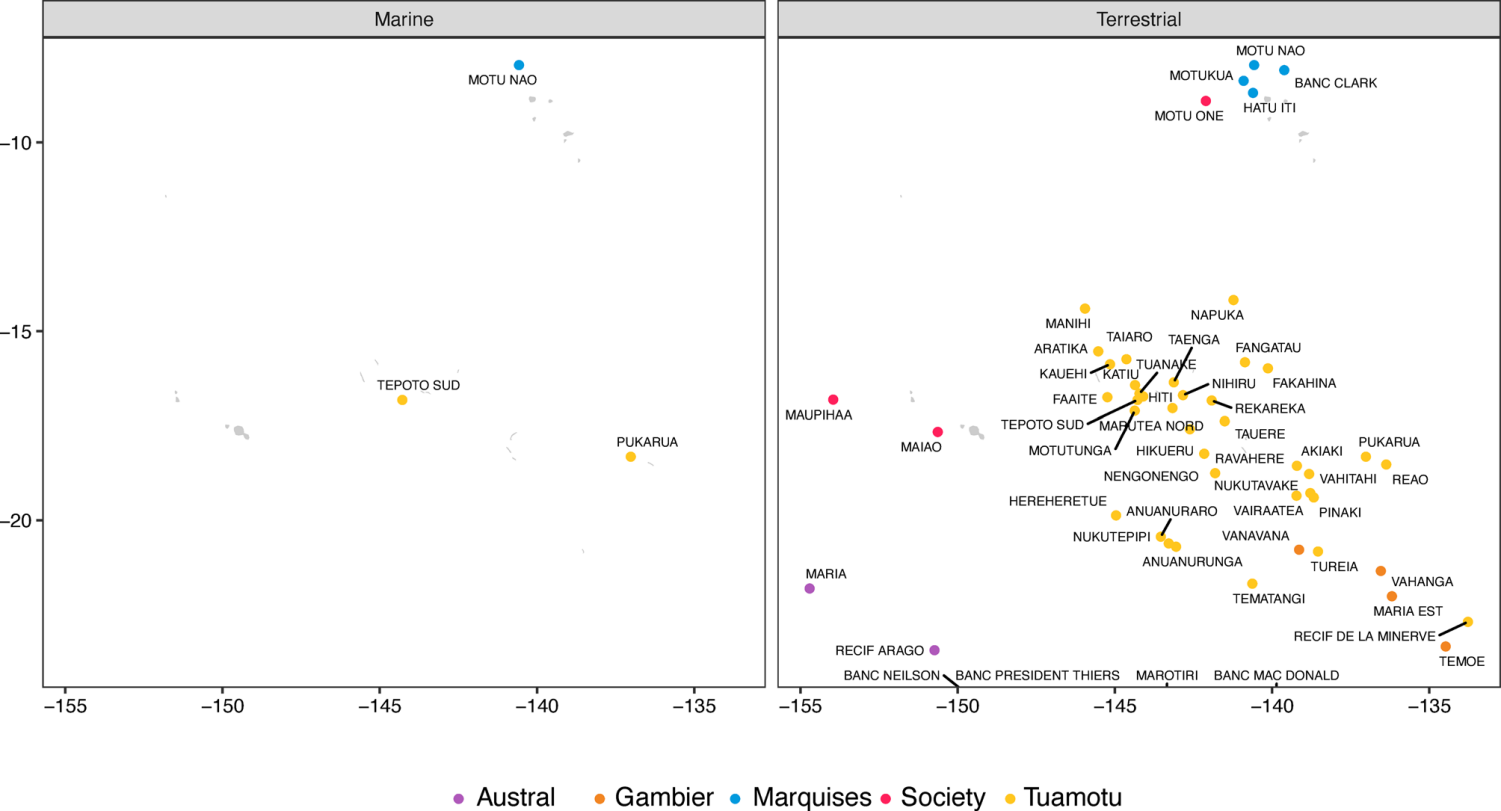
Map of French Polynesian “neglected” islands (i.e., without species records) in marine (left panel) and terrestrial (right panel) datasets. The islands are color-coded according to their respective archipelagos.

Islands for which we were unable to fit SACs were classified as either “neglected islands” (i.e., with no data at all) or “poorly-documented islands” (i.e., with not enough data). For the marine and terrestrial data, we identified three and 52 neglected islands, respectively. The problem of missing data was prevalent across archipelagos, but less important in the Society and Austral archipelagos (Fig. 5). We found 34 and 36 poorly-documented islands for marine and terrestrial ecosystems, respectively. The data scarcity was particularly pronounced in the largest archipelago, the Tuamotu, as well as in the southernmost archipelago, the Gambier (Fig. S4).

### Sampling bias due to human accessibility

**Fig. 6:**
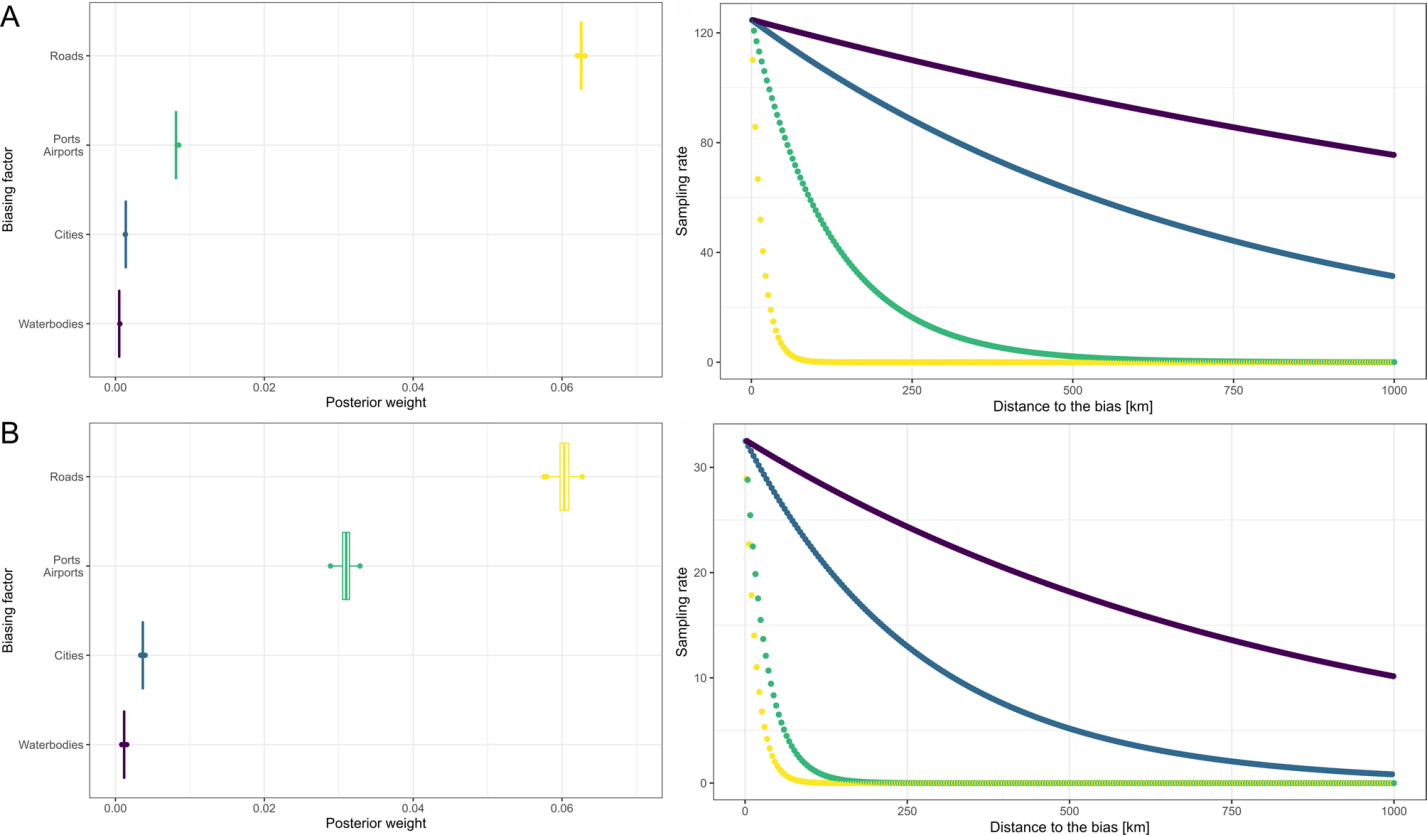
Accessibility bias. Results from the *calculate_bias* function (*Sampbias* package) which estimates marine (**a**) and terrestrial (**b**) expected occurrences based on accessibility, illustrating the impact of various infrastructure types on species sampling efforts. (Left) The posterior weight shows the relative importance of anthropic accessibility factors: roads, ports/airports, cities, and waterbodies—on sampling efforts in both ecosystems. (Right) The sampling rate, i.e., the expected number of occurrences, as a function of distance from accessibility factors is depicted for marine (**a**) and terrestrial (**b**) environments.

Sampling effort for marine species was primarily influenced by proximity to roads (w = 0.063), indicating a strong spatial bias towards areas with developed infrastructure. Airports had a moderate effect on sampling distribution (w = 0.008). In contrast, proximity to cities (w = 0.001) and waterbodies (w = 0.0005) had negligible impacts on sampling intensity (Fig. 6; Table S1).

Similar results were found for the terrestrial data, where the presence of roads contributed the most to the accessibility bias (*w* = 0.060). The effect of airports and ports was moderate (*w* = 0.031) while the influence of cities and water bodies was negligible (cities’ *w* = 0.004, waterbodies’ *w* = 0.001) (Fig. 6). The model also revealed a low number of marine and terrestrial records (Table S1), even after correcting for accessibility biases, in the Tuamotu and Gambier Archipelagos, except for Mangareva, Hao, and Arutua Islands. In contrast, most islands in the Society Archipelago were oversampled relative to the overall sampling effort across French Polynesia.

## Discussion

Our study compiles the most comprehensive open-source database on animal biodiversity in French Polynesia, illuminating regional- and island-scale biodiversity patterns of marine and terrestrial fauna across this vast and fragmented territory. While our results highlight significant disparities in sampling effort across islands, this work offers valuable quantitative insights into completeness of taxonomic and spatial data throughout French Polynesia. This work also highlights understudied areas and taxonomic groups, providing a practical tool for conservation planners to guide future sampling strategies and enhance biodiversity representativeness. We argue that this integrative approach is essential for explicitly addressing the inherent biases often present in large-scale biodiversity studies^23^.

### Building an accurate open-source biodiversity dataset

While open-source biodiversity datasets offer unique opportunities for studying macroecological processes, global repositories face criticism due to significant variation in data quality and quantity, depending on geographic, temporal, and taxonomic factors^22^. Ignoring these caveats can lead to erroneous conclusions. However, when carefully considered, they can enhance the utility of open-source data by highlighting critical biodiversity knowledge gaps (e.g^80–82^). Addressing uncertainties in the data first requires acknowledging that open-source biogeographic datasets are likely to be incomplete^25^, especially in vast and fragmented regions and for specific groups of organisms. Secondly, standardised taxonomic repositories (e.g., WORMS) offer workflows for cleaning data retrieved from open-source platforms while adhering to FAIR data-sharing principles. Here, by applying previously validated filtering protocols^63, ^we enhanced the geographic and taxonomic accuracy of GBIF records for French Polynesia, closely matching recent expert taxonomic assessments.

Our database contains a total of 7,188 species, including 1,893 vertebrates and 5,295 invertebrates. Regarding vertebrates, we found that every known marine mammal (26 out of 26 species) and a large number of birds (126 out of 175 species) previously documented in the region are represented^83^. Our database includes 2,552 marine molluscs out of 3,022 referenced in a recently published checklist and identification guide^46^, and the Teleostei class included 1,547 species, which is more than the 1,310 reported in the most complete identification guides for the region^84,85^. While the taxonomic coverage is reassuring for marine species, it remains relatively limited for terrestrial species. For example, our records include only 757 out of 2,497 insect species (Insecta) and 63 out of 365 spider species (Arachnida) described in the region^49^. Data scarcity for insects is a global issue, and in some regions, it is partly driven by species extinction rates that outpace discovery rates^86,87^. Islands, which harbour approximately 20% of the world’s terrestrial biodiversity, are critical reservoirs of fragile and threatened biodiversity^56^. This highlights the urgent need to document the exceptional biodiversity of insular countries like French Polynesia, where some taxonomic groups, such as ground beetles, contribute significantly to global biodiversity^56,88^. Our study provides an efficient framework for identifying poorly sampled species, which can be extended to other taxonomic groups in French Polynesia (e.g., plants or algae) and applied more broadly to other regions.

### Linnean shortfall

The Linnean shortfall—i.e., only a fraction of the planet’s species has been described—is a major gap in our understanding of biodiversity^18^, limiting our ability to effectively address the ongoing extinction crisis^2^. The Linnean shortfall is partly driven by taxonomic sampling biases, where societal preferences influence which groups are more frequently recorded^21^. This explains why patterns of sampling efforts are often represented by homogeneously-sampled taxonomic groups such as marine mammals^82^, fishes^89^ or insects^90^. Notably, our taxonomic bias analysis revealed a significant under-representation of non-charismatic invertebrate species such as Gastropoda, Malacostraca, Anthozoa, Bivalvia, Polychaeta in the marine environment, as well as Insecta, Gastropoda, Arachnida, Malacostraca, in terrestrial ecosystems. This finding aligns with Troudet et *al.* (2017)^21^, who also identified biases against these classes at the global scale. Conversely, vertebrates were well-represented, with the humpback whale (*Megaptera novaeangliae*) being one of the most frequently recorded species. This discrepancy often stems from the aesthetic appeal of certain species, which influences both public interest and scientific focus^91–93^. Furthermore, studies have effectively shown that visual appeal shapes the perception and prioritisation of species in research and conservation^93^. To address these biases and enhance biodiversity inventories in French Polynesia, our dataset can help guide future research priorities, focusing on the underrepresented invertebrates and terrestrial species identified. By addressing these gaps, we can move towards a more comprehensive and balanced understanding of biodiversity, which is crucial for developing effective conservation strategies.

### Wallacean shortfall

Another significant gap in our understanding of biodiversity is the incomplete knowledge of species’ geographic distribution, also known as the Wallacean shortfall^25,94^. Despite extensive efforts, biodiversity sampling remains a resource-intensive, time-consuming and costly process, often resulting in substantial gaps in the spatial coverage of species records. Short-term projects frequently fail to capture the full spectrum of species within an assemblage because many species can be cryptic, rare or elusive, ultimately leading to incomplete assessments of global biodiversity patterns. However, these data gaps and uncertainties can be gauged and possibly mitigated through robust modelling approaches^23^. In our study, marine inventory completeness was consistently moderate across French Polynesia’s archipelagos, being up to 74% of known species at the regional scale. Furthermore, none of the species accumulation curves for the archipelagos reached saturation, indicating that species richness predictions require more sampling to improve accuracy. Statistical methods to correct these biases (e.g.,^66^), could be used for comparing community assemblages among archipelagos, as has been recently done with woody plants^95^. Another strategy is to focus on well-documented groups, with complete inventories, enabling the description of their spatial distribution patterns^96^.

For terrestrial species, we found that inventory completeness was more variable than that of marine species. The Marquesas Archipelago was especially under-surveyed, as only half of the total estimated animal species have been documented. Owing to their geographical isolation and intricate topography, the Marquesas Islands harbour a high level of floral and faunal endemism, with many native and endemic arthropod species probably yet to be discovered^55^. Indeed, many studies have highlighted the uniqueness of this archipelago in terms of species assemblages^43,97^ and genetic diversity^98^. This biological distinctiveness, combined with the underrepresentation of terrestrial studies compared to marine ones, likely accounts for the discrepancy with other archipelagos, despite the strong interest that scientists have expressed for this biodiversity hotspot^99^. Prioritising terrestrial biodiversity research in the Marquesas is crucial for establishing reliable comparisons across the land-to-sea continuum in this archipelago. Similarly, a more sustained sampling effort is much needed in the Gambier and Tuamotu Archipelagos, where a significant number of islands remain insufficiently inventoried. This is an urgent call because, while scientific expeditions could potentially discover new species (e.g.,^100^), other species could become extinct before being documented (e.g^101,102^).

Sampling effort biases can obscure the true spatial distribution of biodiversity, complicating the identification of biodiversity hotspots and the quantification of biodiversity loss^103^. Decision-makers rely on data to inform and justify their political choices. However, gaps in biodiversity inventories can hinder conservation efforts and limit our ability to assess their effectiveness. For instance, if the distribution of an endangered species is poorly documented, it becomes difficult to identify and prioritize areas for protection. In Polynesia, a marine mammal observation network has been established, along with sanctuaries on three islands—Rurutu, Tahiti, and Moorea—contributing to the protection of these threatened species. Nevertheless, other areas also merit consideration for protection due to their high marine mammal diversity. Notably, Raiatea-Taha’a stands out, having recorded the highest number of species (23 species) and a significant number of sightings (104 records). Furthermore, Raiatea and Tahaa, which together form the largest lagoon in the Society Archipelago, may host a particularly high level of biodiversity not fully reflected in current GBIF data. This hypothesis is supported by research showing that 26 of the 32 marine sponges recorded across French Polynesia were found in Raiatea-Taha﻿’a^104^. Similarly, our findings confirmed that the island of Rapa harbours remarkable marine diversity, as evidenced by studies on coral-reef and terrestrial communities, including taxa unique to this island^105,106^. However, despite being one of the best documented islands in the archipelago (*C* = 75.6%), Rapa’s inventory completeness remains behind the global threshold of 80%, suggesting that further sampling efforts are necessary to fully capture this island’s biodiversity. Overall, our study contributes to addressing this gap by pinpointing overlooked locations of the Polynesia-Micronesia biodiversity hotspot.

Conservation science is often compelled to assist in decision-making based on limited and incomplete data^107^. The spatial heterogeneity in sampling effort that we identified for both marine and terrestrial fauna in French Polynesia is considerable, with up to 70% of islands lacking data on their terrestrial environments. This striking data deficiency was also evidenced by another study using GBIF data to analyse species diversity in a remote region^63^. An additional challenge, particularly for vast and fragmented territories such as French Polynesia, is the need for data at a sufficiently high spatial resolution to capture island-wide variation. We identified 52 islands that either lacked digital data entirely or were poorly documented, likely due to their remoteness. To fill the spatial gaps in biodiversity data for French Polynesia, we recommend that future sampling efforts prioritise these islands, while also considering the disparity in data coverage between marine and terrestrial ecosystems.

### The marine-terrestrial sampling bias

Marine and terrestrial ecosystems are often studied separately, partly due to historical, cultural, or practical reasons^108,109^. However, because the land-sea continuum operates as an integrated meta-ecosystem, this research divide hampers our ability to fully understand and effectively protect interconnected ecosystems^103,110^. Maintaining a healthy land-sea ecosystem is particularly crucial in small-island territories, where biodiversity is vulnerable to human activities^35,56^and where the wellbeing of local populations heavily depends on local natural resources, especially through fishing and tourism. French Polynesia is no exception, with tourism as its primary economic activity and fish and invertebrates as staples in the local diet^111^. Unlike the global trend^103^our data show that French Polynesian biodiversity is better documented in marine ecosystems than in terrestrial ones. This discrepancy is partly due to the focus of scientific research and exploration on marine environments (e.g., the oldest of the two major ecology research units in French Polynesia, the CRIOBE, is entirely focused on marine environments) and to the inaccessibility of the mountainous regions^54^ and seamounts^112^. The gap is also likely influenced by the huge difference in surface area between land (4,167 km²) and sea (2.5*10^6^ km²), which may also explain why the marine habitats host 20 times more species than terrestrial ones. While surface-area differences are a factor to consider, our records indicate that the disparity is also driven by a lack of terrestrial data for over 52 islands, compared to just two islands with missing marine data. The observed imbalance in marine versus terrestrial data coverage is not only due to the inherent differences between these ecosystems but also reflects underlying biases in sampling practices, exacerbated by the accessibility factors.

### Sampling bias is partly influenced by accessibility factors

The accessibility bias hypothesis posits that more accessible areas tend to be surveyed more frequently than less accessible zones^79^. This can significantly impact the global understanding of natural communities^103,113^. Our database revealed a pronounced geographic bias in species records, with the most accessible islands (i.e., Tahiti and Moorea in the Society Archipelago, Fakarava in the Tuamotu) being heavily sampled. In contrast, less accessible islands (e.g., Tureia, Napuka and Tenarunga in the Tuamotu, Motu One and Motu Nao in the Marquesas) are poorly documented. However, Rapa Island stands out as an exception, having attracted significant attention from the scientific community due to its hosting of several threatened endemic plant and animal species^54,105,106,114^. The sampling bias in Tahiti and Moorea is also likely related to the presence of local research institutions (e.g., CNRS-EPHE-Université de Perpignan CRIOBE station, Ifremer, IRD, UC-Berkeley Gump station, University of French Polynesia) there. While Tahiti’s international airport contributes to the sampling bias observed in the Society Islands, our accessibility bias analysis indicated that the distance from ‘airports and ports’ was not the main anthropogenic factor explaining the variance in sampling effort at the scale of French Polynesia. Overall, our accessibility bias analysis showed that sampling efforts in both marine and terrestrial datasets are predominantly skewed towards areas near roads and, to a lesser extent, airports/ports. This aggregation pattern around roads is well-documented in the literature for both terrestrial and marine species^103,115^ particularly in studies based on citizen-science data^116^.

Accessibility biases can vary depending on geographic and taxonomic contexts^116^ highlighting the importance of considering situations on a case-by-case basis. For instance, Freitag et al. (1998)^117^ found that records of smaller species in African terrestrial ecosystems were minimally affected by accessibility biases, whereas larger species were disproportionately represented in protected areas. Similarly, Cardoso et al. (2024)^118^ identified various accessibility-bias factors for marine species in the western Atlantic Ocean, including proximity to the coastline, research institutions, ports, protected areas, and urban centres. Recognizing and understanding the nuances underlying these various biases is crucial for enhancing the accuracy and comprehensiveness of biodiversity datasets.

### Institutional bias in open-source databases

While accessibility factors provide important insights into sampling patterns, they are not the sole source of bias impacting our biodiversity records. Institutional biases, particularly those associated with open-source databases, might also play a crucial role. The unevenness in data contributions often stems from disparities in funding, data-sharing policies, and digitization efforts across different regions and institutions. The soaring popularity of GBIF data worldwide is reflected in our dataset for French Polynesia, where the number of records per year increased from 10 in 1950 to 1,866 in 2022. We anticipate that the dataset will continue to grow with the engagement of additional contributors, thereby enhancing its reliability^119^ if institutions continue to adhere to standardisation protocols^10^. Interestingly, the surge in data during 2006, 2011, and 2012, which constitutes the bulk of the dataset, was driven by the digitization of the French Museum of Natural History dataset (managed by PatriNat) and a major field sampling campaign by Cornell University (USA). The patchiness in data contributions to global open-source databases can be attributed to differences in funding and data-sharing policies across countries, inadequate efforts in digitalising local and national databases, and the sporadic and spatially heterogeneous nature of formal research campaigns^26^. Combining GBIF records with national databases can yield more complete inventories, as demonstrated by De Araujo et al. (2022)^75^ for Amazonian epiphytes. In our study, we applied this approach by not only relying on GBIF as the primary data source but also integrating nine additional local datasets to enhance the completeness of our inventory. This selective integration of external data, including local sources, helped reduce coverage gaps while maintaining data quality, underscoring the importance of leveraging both global and local data sources to mitigate biases in biodiversity records. In the case of French Polynesia, engaging local research institutions, private entities, government agencies and developing a citizen science network to compile and share existing (but often inaccessible) information would significantly reduce biases and strengthen the database. The use and adaptation of existing portals such as FauneFrance (https://www.faune-france.org/) or iNaturalist (https://www.inaturalist.org/) to local flora and fauna could for example be advocated to further centralise and favour the collection and compilation of local naturalist data.

### Capitalising from citizen science while reducing biases in open-source datasets

Addressing biases and shortfalls in open-source biodiversity datasets is crucial to ensure their efficiency and accuracy in describing species distribution patterns. Citizen science has been increasingly recognized as an effective method for filling gaps in biodiversity information, especially in areas where formal scientific campaigns are limited or sporadic^14,120^. In our database for French Polynesia, we observed an increase in species records driven by citizen science initiatives, in agreement with the global trend^9^. Indeed, a substantial 21.7% of records originated from participatory science efforts. While citizen scientists may not always adhere to standard scientific protocols, their contributions provide valuable insights into broader trends, which can then be rigorously analysed. To minimise taxonomic and geographic biases, the involvement of taxonomic experts remains crucial^62^.

### Conclusions and perspectives

Centralising biodiversity information from museums, research institutions, and citizen scientists into big-data platforms offers a transformative opportunity for evaluating species biodiversity in understudied regions. These platforms enable comprehensive data analysis, facilitate global collaboration, engage the public in science, and ultimately contribute to more informed conservation strategies and biodiversity management. Our study provides significant insights into the biodiversity patterns of both marine and terrestrial fauna across the vast and fragmented territory of French Polynesia. We found that while marine inventory completeness is relatively high, averaging up to 76% of known species at the regional scale, terrestrial biogeography remains underexplored (average of 65%), particularly in the Marquesas and Gambier Archipelagos. The analysis indicates a notable skew in the data toward specific taxonomic groups, highlighting the urgent need for comprehensive surveys to fill these gaps. Furthermore, our findings underscore the value of citizen science initiatives, demonstrating their potential to enhance biodiversity knowledge in regions where formal scientific efforts are limited. Overall, this research not only emphasises the richness of biodiversity in French Polynesia but also calls for collaborative efforts to centralise and analyse biodiversity data. These efforts are crucial for aiding in conservation strategies and improving management of the unique ecosystems in the Indo-Pacific region, a global biodiversity hotspot that includes Micronesia, Polynesia, and Fiji^121^. By providing a reliable, spatially resolved biodiversity dataset, this study lays the foundations for future macroecological research in French Polynesia that will help respond to both fundamental and applied environmental questions.

## Electronic supplementary material

Below is the link to the electronic supplementary material.

Supplementary Material 1

Supplementary Material 2

## Data Availability

The analyses scripts are available in GitHub (https://github.com/KilianBARREIRO/biogeography_datadiv). The data are available in SEANOE (https://www.seanoe.org/data/00878/99018/).
