# Supplementary material for: Species richness variation in marine and terrestrial fauna across widespread, fragmented territories: assessing inherent challenges of data scarcity at local and regional scales
Source: Sci Rep. 2025 Jul 1;15:21043. doi: 10.1038/s41598-025-06631-4 (PMC12215423; doi:10.1038/s41598-025-06631-4)
Supplement: Supplementary file 1 — Supplementary Material 1 [file 41598_2025_6631_MOESM1_ESM.docx]

**Supplementary Material**

**Table S1.** Bias posterior weights (mean ± SD) showing the relative importance of anthropic accessibility factors (roads, ports/airports, cities, and waterbodies) on sampling efforts in both marine and terrestrial ecosystems.

|  | Marine | Terrestrial |
| --- | --- | --- |
| Cities | 0.00138 ± 0.00002 | 0.00368 ± 0.00008 |
| Roads | 0.0626 ± 0.00017 | 0.0603 ± 0.00086 |
| Waterbodies | 0.00050 ± 0.00002 | 0.00117 ± 0.00012 |
| Airports | 0.00813 ± 0.00009 | 0.0310 ± 0.00066 |


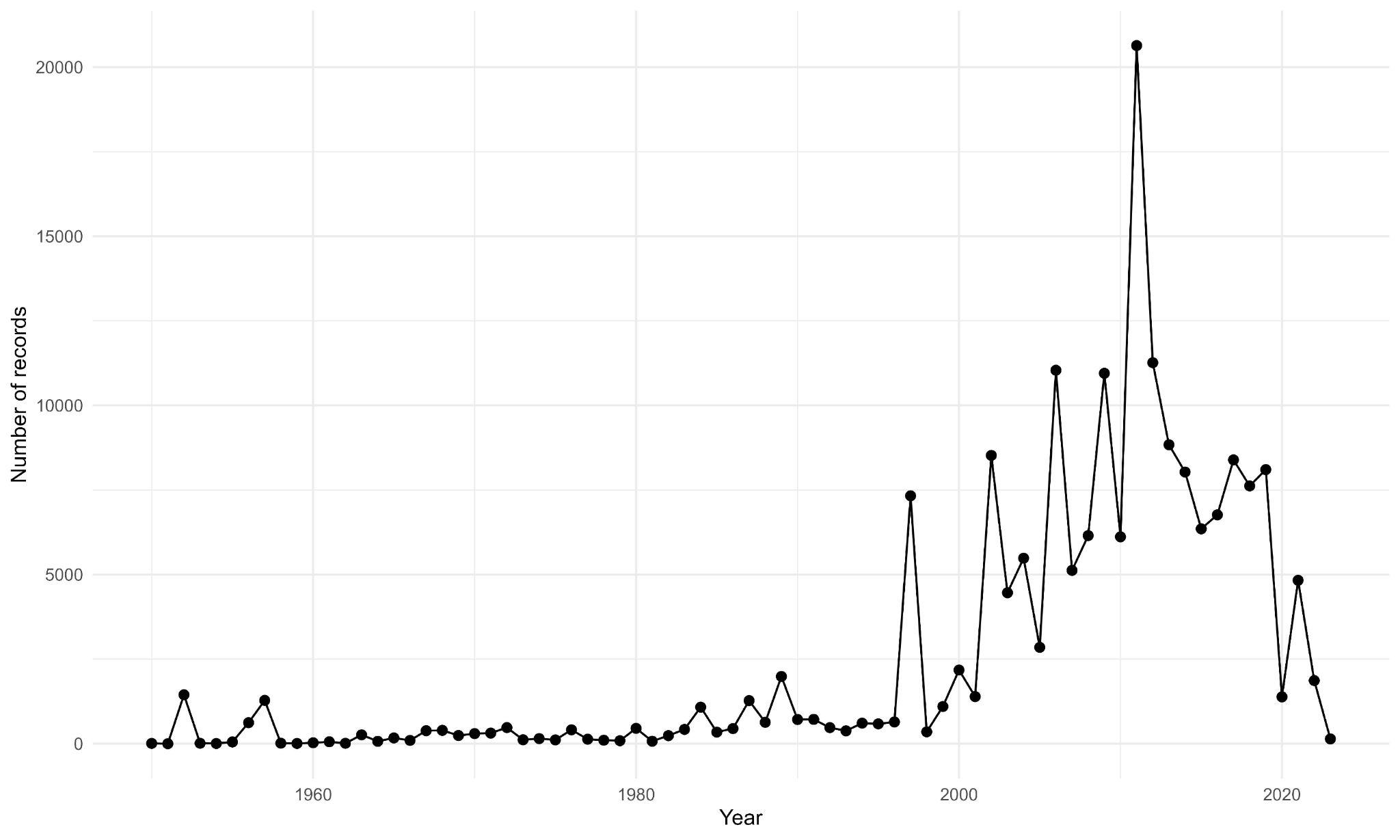


**Figure S1.** Number of GBIF (Global Biodiversity Information Facility) records for fauna in French Polynesia between 1950 and 2023.


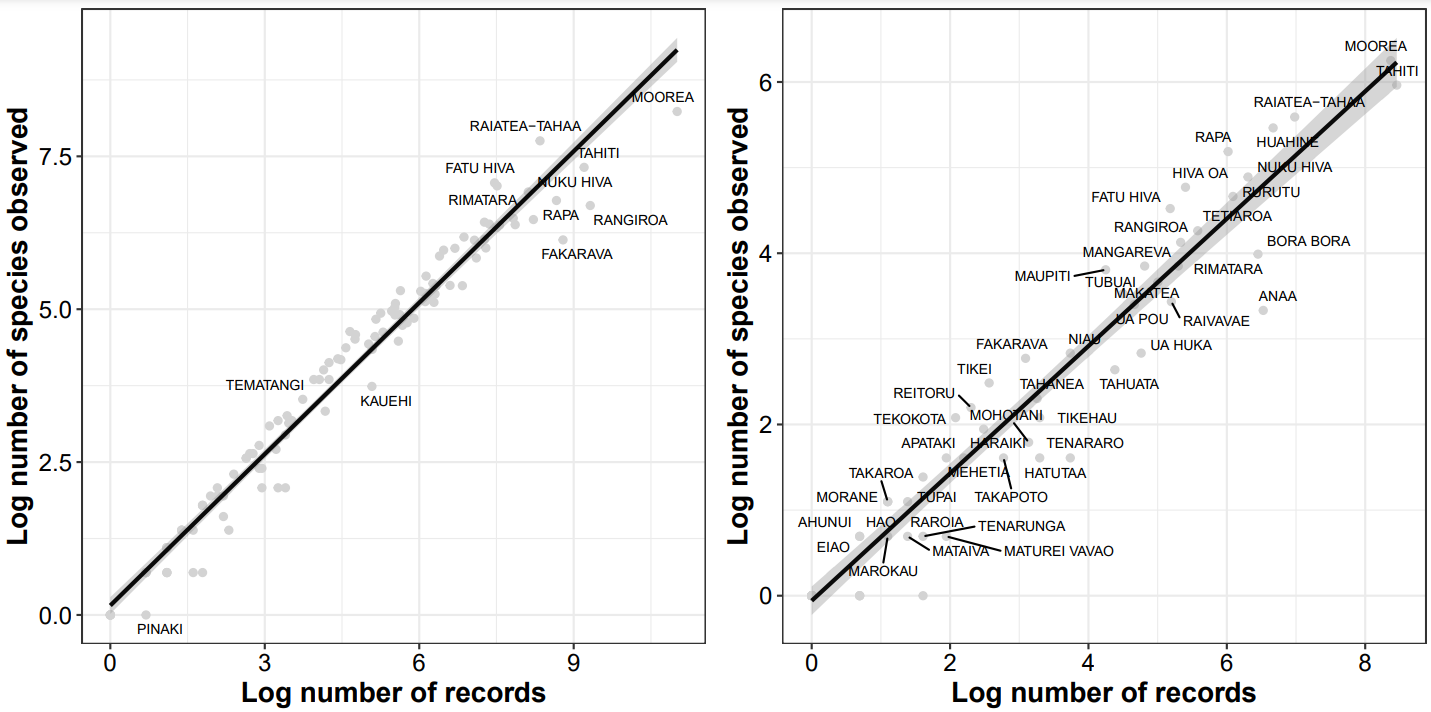


**Figure S2.** Record bias. Correlation between the number of species per island and records across French Polynesia for marine (left panel) and terrestrial (right panel) habitats.


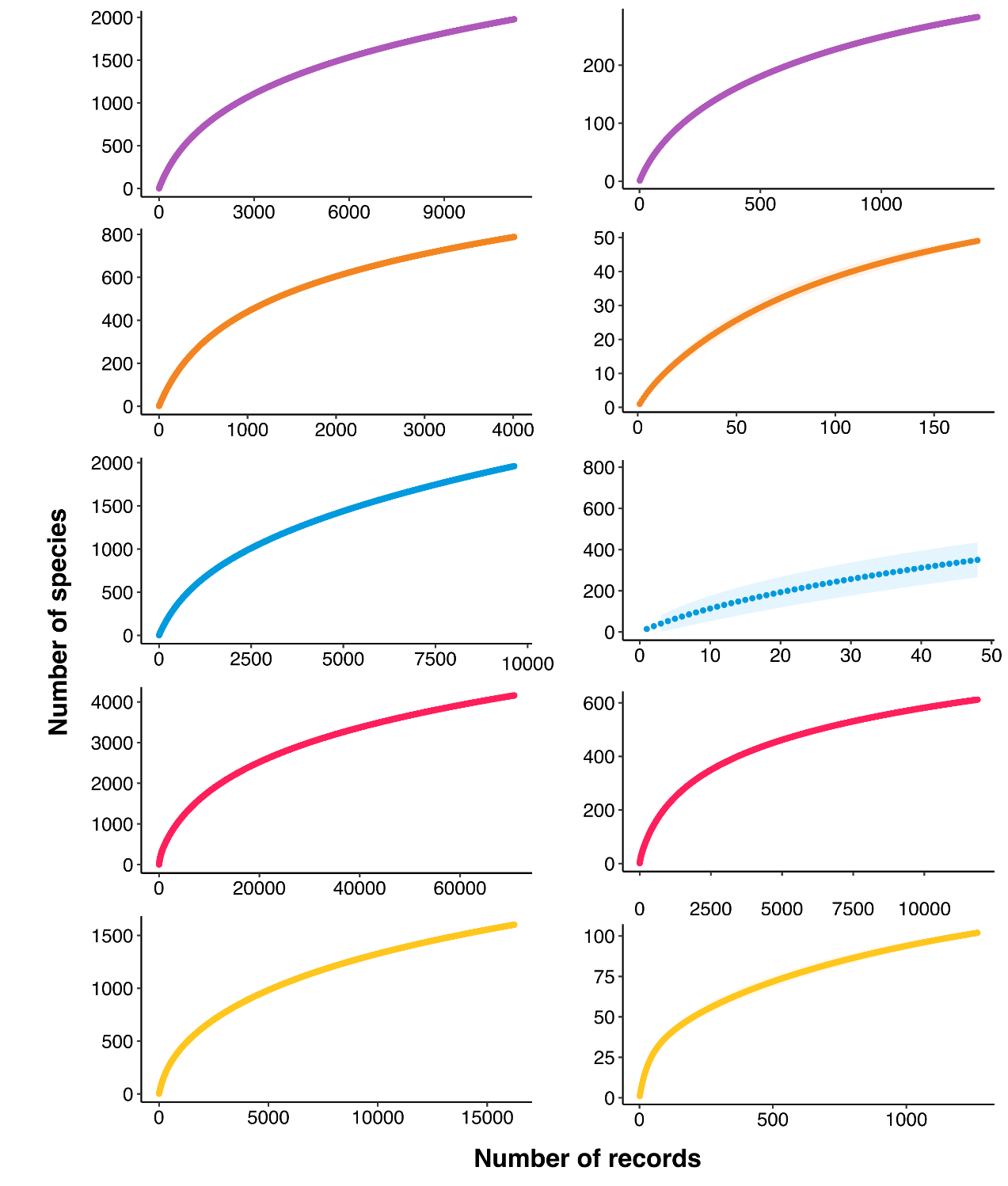


**Figure S3**. Species Accumulation Curves produced from resampling. Modelled relationships between the number of species and the number of records for marine (left panels) and terrestrial (right panels) habitats. The lines represent Michaelis-Menten model fits, each archipelago is represented by a specific colour (purple: Austral, orange: Gambier, blue: Marquesas, pink: Society, yellow: Tuamotu), and the shaded zone illustrates 95% confidence intervals. The half-saturation constant (K), representing the area required to capture 50% of the expected species, is shown with vertical dashed lines.

**
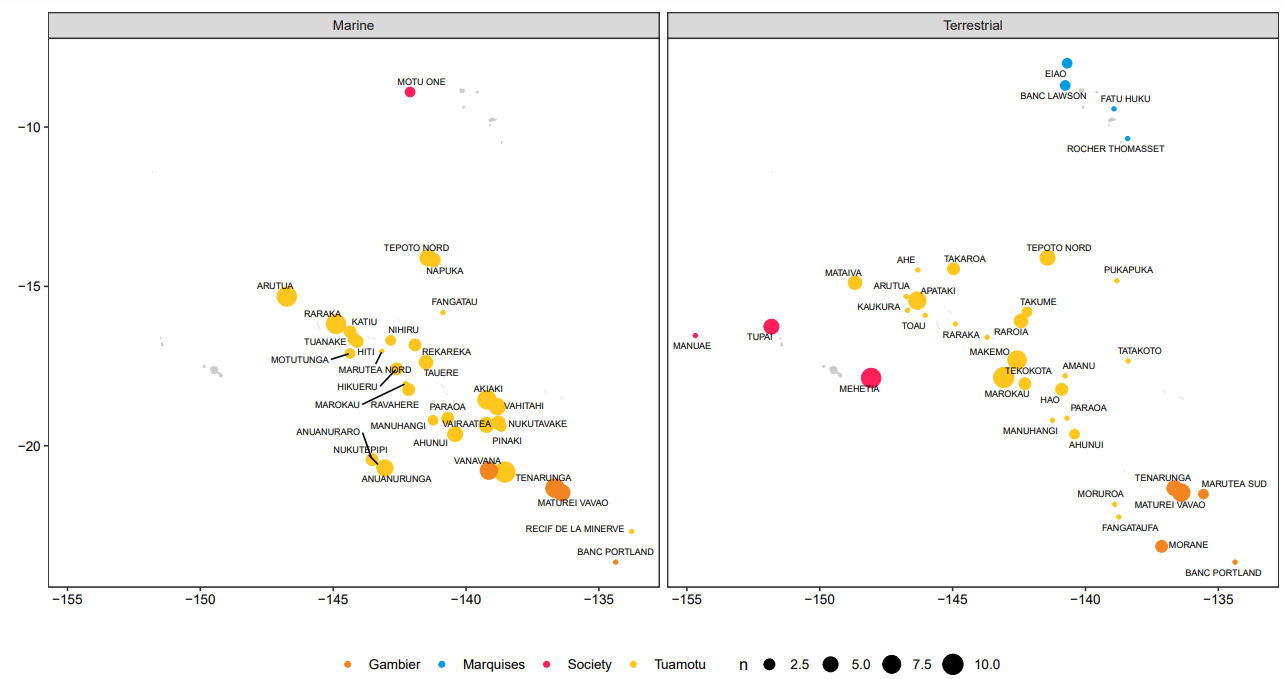
**

**Figure S4.** Poorly-documented French Polynesian islands for marine (left) and terrestrial (right) ecosystems. The number of records for these islands is represented by variable point sizes (1 to 10 records). Islands are colour-coded according to their respective archipelagos.
