# Supplementary material for: Species richness variation in marine and terrestrial fauna across widespread, fragmented territories: assessing inherent challenges of data scarcity at local and regional scales
Source: Sci Rep. 2025 Jul 1;15:21043. doi: 10.1038/s41598-025-06631-4 (PMC12215423; doi:10.1038/s41598-025-06631-4)
Supplement: Supplementary file 2 — Supplementary Material 2 [file 41598_2025_6631_MOESM2_ESM.docx]

**Table S2.** List of references (including GBIF, local French Polynesian reference guides, expedition reports, and data repositories) consulted to build a species occurrence dataset for French Polynesia. Year: Year of publication or release of the data source; Title: Title of the article, database, expedition, or report considered for potential data merging; Authors: List of authors or contributors of the source; Habitat: Type of habitat concerned (e.g., Marine, Terrestrial, Freshwater); Source_type: Nature of the source (e.g., peer-reviewed article, report, database); Issue_type: Data-specific issue addressed (e.g., lack of geospatial information); Data_merged: Indicates whether the data were included in the dataset before the curation and cleaning process (Yes/No); Available_data: Indicates whether the data was accessible to download (Yes/No); Spatial_resolution: Spatial resolution or extent (e.g., coordinates, region level); Repository: Where the data are stored or accessible (e.g., Zenodo, Dryad, URL); Notes: Contains specific notes or additional details related to the entry, such as particular methodological considerations, data limitations, or clarifications.

| Year | Title | Authors | Habitat | Source_type | Issue_type | Data_merged | Available_data | Spatial_resolution | Repository | Notes |
| --- | --- | --- | --- | --- | --- | --- | --- | --- | --- | --- |
| 2025 | A seascape dichotomy in the role of small consumers for coral reef energy fluxes | Brandl et al. | Marine | article | None | YES | YES | Coordinates | <https://doi.org/10.6084/m9.figshare.28400828.v1> | 778 Occurrences (insects, worms, molluscs, or small vertebrates) cryptobenthic communities data in 2017/2018 |
| 2024 | Mesophotic coral ecosystems of French Polynesia are hotspots of alpha and beta generic diversity for scleractinian assemblages | Perez-Rosales G. et al. | Marine | article | Missing or invalid geospatial data | NO | NO | None | <https://datadryad.org/dataset/doi:10.5061/dryad.rjdfn2zc7> | Transects, no geospatial data |
| 2023 | Ocean Biodiversity Information System | OBIS | Marine | website | None | YES | YES | Coordinates | [obis.org](http://obis.org) | GBIF contains the data from OBIS for FP at the time of downloading. |
| 2023 | Global Biodiversity Information System | GBIF | Terrestrial | website | None | YES | YES | Coordinates | [gbif.org](http://gbif.org) | 297,789 Occurrences at the time of downloading (May 2023) |
| 2023 | Diversity, systematics and biogeography of French Polynesian <i>Lobophora</i> (Dictyotales, Phaeophyceae) | Vieira C. et al. | Marine | article | Missing or invalid taxonomical data | NO | NO | Coordinates | <https://www.tandfonline.com/doi/full/10.1080/09670262.2022.2092216?src=> | Non-significant taxonomy (seaweed) |
| 2023 | Pacific Data HUB | CPS | Marine & Terrestrial | website | Missing or invalid geospatial data | NO | NO | None | <https://pacificdata.org/> | Possibly useless, indeed no geospatial data (updated 2025/04/16) |
| 2023 | Citizen science provides valuable data to evaluate elasmobranch diversity and trends throughout the French Polynesia�s shark sanctuary | Seguigne C. et al. | Marine | article | None | YES | YES | Coordinates | <https://doi.org/10.5061/dryad.z08kprrhm> | A dataset about sharks in French Polynesia extracted from the ORP citizen science network: the final dataset has 5 missing shark species and 21,357 missing occurrences (but the data isn't cleaned). It is available early in 2023 and is biased towards the most frequented diving sites. |
| 2021 | Checklists of endemic tracheophytes and bryophytes to the French Overseas Territories | Veron, S. et al. | Terrestrial | article | Missing or invalid taxonomical data | NO | NO | None | <https://doi.org/10.5061/dryad.xpnvx0kfd> | Plants and non-geospatial data |
| 2020 | The Turtle Research and Monitoring Database System | TREDS | Marine | website | Unpublished or unavailable data | NO | YES | Zone or field ID | <https://www.sprep.org/thetreds> | Undownloadable data, encountering sites available between 1970 and 2018 (no coordinates, Cit. Sc) |
| 2019 | A DNA barcode reference library of French Polynesian shore fishes | Delrieu-Trottin E. et al. | Marine | article | Missing or invalid geospatial data | NO | YES | Field ID | <https://www.nature.com/articles/s41597-019-0123-5#Ack1> | No Lat/Lon data but Voucher data from the Smithsonian Institution (~9,200 occurrences in our dataset) |
| 2019 | Global repository for mammal biological data | MaNIS | All | website | Missing or invalid geospatial data | NO | YES | Coord. Ranges | <http://www.manisnet.org/> | Spatial data, but in a range map format |
| 2019 | Resolbing the depth zonation in reef-building corals | Roberts et al. | Marine | article | Missing or invalid geospatial data | NO | YES | Field ID | <https://doi.org/10.1002/ecy.2761> | NA |
| 2017 | Checklist of the terrestrial and freshwater arthropods of French Polynesia (Chelicerata, Myriapoda, Crustacea, Hexapoda) | Ramage T. | Terrestrial & Freshwater | article | Missing or invalid geospatial data | NO | NO | None | <https://sciencepress.mnhn.fr/en/periodiques/zoosystema/39/2/liste-de-reference-des-arthropodes-terrestres-et-d-eau-douce-de-polynesie-francaise-chelicerata-myriapoda-crustacea-hexapoda> | Original references/sources provided in the Appendix, 3,030 species listed (officially 2,995 at species level, subspecies have been smashed during duplicates cleaning, all listed species are present in taxref (see paper methods)) |
| 2017 | Biogéographie des mollusques marins de Polynésie française | Salvat B, Trondle J. | Marine | article | Unpublished or unavailable data | NO | YES | Atoll, island or archipelago | <https://www.researchgate.net/publication/327780165_Biogeographie_des_mollusques_marins_de_Polynesie_francaise> | Unpublished |
| 2017 | Birds of Eastern Polynesia - a biogeographic atlas | Thibault J.C. et Cibois A. | Terrestrial | book | Missing or invalid geospatial data | NO | YES | Zone or field ID | personal_copy | Marine birds are included, but mostly provide their breeding site distribution, not the at-sea distribution. |
| 2016 | Biodiversite terrestre et marine des iles Marquises, Polynesie francaise | Galzin R., Duron S.-D., Meyer J.-Y. (eds) | Marine & Terrestrial | book | Missing or invalid geospatial data | NO | YES | Atoll, island or archipelago | personal_copy | NA |
| 2015 | Environnement marin des îles Australes, Polynésie Française | Salvat T. et al. | Marine | ebook | Unpublished or unavailable data | NO | NO | None | <http://www.ircp.pf/environnement-marin-des-iles-australes-polynesie-francaise-le-livre/> | NA |
| 2015 | Global repository for fish biological data | Fishnet2 | Marine & Freshwater | website | None | YES | YES | Atoll, island or archipelago | <http://www.fishnet2.net/> | 2,790 Occurrences. |
| 2014 | Terrestrial biodiversity of the Austral islands, French Polynesia | Meyer J.Y. et Claridge E.M. | Terrestrial | book | Unpublished or unavailable data | NO | NO | None | personal_copy | NA |
| 2013 | Bioclimatic and physical characterization of the world’s islands | Weigelt P. et al. | Terrestrial | article | Missing or invalid taxonomical data | NO | YES | Coordinates | <https://www.pnas.org/doi/pdf/10.1073/pnas.1306309110> | No biodiversity data (no taxonomy). |
| 2013 | Expedition TUHAA PAE | Consortium | Marine | program | None | YES | YES | Coordinates | <https://expeditions.mnhn.fr/campaign/tuhaapae2013> | available on MNHN Website (Australs) |
| 2011 | Expedition PAKAIHI TE MOANA | Consortium | Marine | program | None | YES | YES | Coordinates | <https://expeditions.mnhn.fr/campaign/pakaihiitemoana> | available on MNHN Website (Marquesas) |
| 2010 | Guide des crustacés décapodes du Pacifique Sud | Poupin J. et Juncker M. | All | ebook | Missing or invalid geospatial data | NO | YES | Atoll, island or archipelago | <https://www.spc.int/DigitalLibrary/Doc/FAME/Reports/CRISP/ENG_FR_2010_Guide_decapod_crustaceans.html> | provides a species list with only broad info on their distribution ( https://doi.org/10.5479/si.00775630.451.1) |
| 2009 | Dominant benthic mollusks in closed atolls, French Polynesia | Salvat B. | Marine | article | Unpublished or unavailable data | NO | YES | Atoll, island or archipelago | <https://www.jstage.jst.go.jp/article/galaxea/11/2/11_2_197/_pdf> | Unpublished or inaccessible, Island/Atoll level coordinates |
| 2009 | Diversity of Coralliophilinae (Mollusca, Neogastropoda, Muricidae) at Austral Islands (South Pacific) | Oliverio M. | Marine | article | Missing or invalid geospatial data | NO | YES | Coord. Ranges | <https://www.researchgate.net/publication/232668650_Diversity_of_Coralliophilinae_Mollusca_Neogastropoda_Muricidae_at_Austral_Islands_South_Pacific> | The article is based on personal/unpublished data and provides range lat/lon data rather than specific coordinates. |
| 2009 | Inventory of Marine Molluscs of French Polynesia | Trondle J., Boutet M. | Marine | report | Missing or invalid geospatial data | NO | NO | None | <https://repository.si.edu/bitstream/handle/10088/8009/ARB_570_Trondle_etal.pdf?sequence=1&isAllowed=y> | Checklist without geospatial data |
| 2009 | Expedition TARASOC | Consortium | Marine | program | None | YES | YES | Coordinates | <https://expeditions.mnhn.fr/campaign/tarasoc> | available on MNHN Website (Society) |
| 2008 | Biogeography of the fauna of French Polynesia: diversification within and between a series of hot spot archipelagos | Gillespie R.S. et al. | Terrestrial | article | Unpublished or unavailable data | NO | NO | None | <https://www.researchgate.net/publication/23248424_Review_Biogeography_of_the_fauna_of_French_Polynesia_Diversification_within_and_between_a_series_of_hot_spot_archipelagos> | Unpublished |
| 2008 | Coralliophilinae (Neogastropoda: Muricidae) from the Marquesas Islands | Oliverio M. | Marine | article | NA | NO | NA | NA | <https://www.researchgate.net/publication/235631037_Coralliophilinae_Neogastropoda_Muricidae_from_the_Marquesas_Islands> | Data from MUSOSTORM 9 (see below in MUSOSTORM 9 Expedition) |
| 2008 | Fishbase | Froese, R. & D. Pauly | Marine & Freshwater | website | Missing or invalid geospatial data | NO | NO | None | <https://www.fishbase.se> | Fish checklist endorsed by OBIS and so present in GBIF originally |
| 2006 | Spatial patterns of benthic invertebrate assemblages within atoll lagoons: importance of habitat heterogeneity and considerations for marine protected area design in French Polynesia | Pante E., et al. | Marine | article | Missing or invalid geospatial data | NO | YES | Atoll, island or archipelago | <https://hal.science/hal-00935865/document> | No Lat/Lon data |
| 2006 | Diversity of coral reef fish assemblages: Modelling of the species richness spectra from multi-scale environmental variables in the Tuamotu Archipelago (French Polynesia) | Mellin C., et al. | Marine | article | Unpublished or unavailable data | NO | NA | NA | <https://reader.elsevier.com/reader/sd/pii/S0304380006002419?token=212D7F18384F0BEE5F811668C7F83BFE21EBD183C7ED5D9D61EC97C71F4960C87B4955E35D7FA24BD93E046BE4B5E347&originRegion=eu-west-1&originCreation=20230510214451> | Data part of Typatoll program (unavailable) |
| 2006 | AquaMaps (Computer-generated predictions of the natural occurrence of marine species) | Kaschner, K. et al. | Marine | website | Unpublished or unavailable data | NO | NA | NA | <https://www.aquamaps.org> | Website not working (contact gien) |
| 2006 | French Polynesia Fish Barcoding Database (Fishbard) | CRIOBE | Marine | website | Unpublished or unavailable data | NO | NO | None | <http://fishbardb.criobe.pf/> | Undownloadable data |
| 2005 | Detecting the effects of natural disturbances on coral assemblages in French Polynesia: A decade survey at multiple scales | Adjerou M., et al. | Marine | article | Unpublished or unavailable data | NO | NA | NA | <https://hal.science/hal-00093846/document> | Unpublished |
| 2005 | Pelagic seabird surveys in the Tuamotu and Gambier archipelagos, FP | Vanderwerf, E. et al. | All | article | Unpublished or unavailable data | NO | YES | Atoll, island or archipelago | NA | Undownloadable data |
| 2002 | Expedition RAPA 2002 | Consortium | All | program | None | YES | NA | NA | <https://expeditions.mnhn.fr/documents/RAPA2002/rapport_Rapa.pdf> | available on MNHN Website (Australs) |
| 2002 | Expedition BENTHAUS | Consortium | Marine | program | None | YES | YES | Coordinates | <https://expeditions.mnhn.fr/campaign/benthaus> | available on MNHN Website (Australs) |
| 2001 | Mass mortality of macrobenthic communities in the lagoon of Hikueru atoll (French Polynesia): | Adjerou M., et al. | Marine | article | Missing or invalid geospatial data | NO | YES | Atoll, island or archipelago | <https://link.springer.com/article/10.1007/PL00006962> | NA |
| 2000 | Physical factors of differentiation in macrobenthic communities between atoll lagoons in the Central Tuamotu Archipelago (French Polynesia) | Adjerou M., et al. | Marine | article | Unpublished or unavailable data | NO | NA | NA | <https://repository.si.edu/bitstream/handle/10088/4796/00415.01.pdf> | No biodiversity data (no taxonomy) nor geospatial data, Unpublished |
| 1998 | Objectives and background to the 1994 Franco-Australian expedition to Taiaro Atoll (Tuamotu Archipelago, French Polynesia) | Galzin R. et al. | Marine | article | Missing or invalid geospatial data | NO | YES | Atoll, island or archipelago | <https://link.springer.com/content/pdf/10.1007/s003380050087.pdf> | Only one island, no coordinates |
| 1998 | Expedition MUSOSTORM 9 | Consortium | Marine | program | None | YES | YES | Coordinates | <https://expeditions.mnhn.fr/campaign/musorstom9> | available on MNHN Website (Marquesas) |
| 1997 | Long-term changes of epibenthic macrofauna communities in a closed lagoon (Taiaro Atoll, French Polynesia): 1972–1994 | Adjerou M. | Marine | article | Missing or invalid geospatial data | NO | NA | NA | <https://link.springer.com/article/10.1023/A:1003052515305> | Unpublished (1972-1994 data about 10 species of molluscs) |
| 1997 | Rapports de mission dans les îles de Polynésie française/French Polynesia islands field-trip reports | Meyer J.Y. | Terrestrial | weblog | Missing or invalid taxonomical data | NO | YES | Atoll, island or archipelago | <http://www.jymeyer.com/article-22992111.html> | Lots of plant inventories, but reports on other taxa might also be useful |
| 1997 | Global Seabird Biological Data | Seabird | Marine | website | Unpublished or unavailable data | NO | NA | NA | <https://www.seabirdtracking.org/> | NA |
| 1996 | Typatoll | Consortium | Marine | program | Unpublished or unavailable data | NO | NA | Atoll, island or archipelago | NA | Unpublished or Private |
| 1994 | Variation in diversity of coral reef fish between French Polynesian atolls | Galzin R. et al. | Marine | article | Missing or invalid geospatial data | NO | YES | Atoll, island or archipelago | <https://link.springer.com/content/pdf/10.1007/BF00301196.pdf> | Islands are unable to be part of a Lat/Lon coordinates dataset |
| 1994 | PART I. ENVIRONMENT AND BIOTA OF THE TIKEHAU ATOLL (TUAMOTU ARCHIPELAGO, FRENCH POLYNESIA) | Intes A., Caillart B. | Marine & Terrestrial | article | Missing or invalid geospatial data | NO | NO | None | <https://repository.si.edu/bitstream/handle/10088/4796/00415.01.pdf?sequence=1> | NA |
| 1993 | Atlas de la Polynésie française. LES PEUPLEMENTS DE POISSONS DES RÉCIFS ET DES LAGONS | ORTSTOM | Marine | atlas | Missing or invalid geospatial data | NO | YES | Atoll, island or archipelago | personal_copy | NA |
| 1993 | Atlas de la Polynésie française. LES PEUPLEMENTS DE MOLLUSQUES | ORTSTOM | Marine | atlas | Missing or invalid geospatial data | NO | YES | Atoll, island or archipelago | personal_copy | NA |
| 1993 | Atlas de la Polynésie française. LES PEUPLEMENTS D'ALGUES | ORTSTOM | Marine | atlas | Missing or invalid geospatial data | NO | YES | Atoll, island or archipelago | personal_copy | NA |
| 1993 | Atlas de la Polynésie française. LES PEUPLEMENTS CORALLIENS | ORTSTOM | Marine | atlas | Missing or invalid geospatial data | NO | YES | Atoll, island or archipelago | personal_copy | NA |
| 1993 | Atlas de la Polynésie française. LA FAUNE TERRESTRE: REPTILES ET MOLLUSQUES | ORTSTOM | Terrestrial | atlas | Missing or invalid geospatial data | NO | YES | Atoll, island or archipelago | personal_copy | NA |
| 1993 | Atlas de la Polynésie française. LA BIOGÉOGRAPHIE RÉCIFALE ET LAGONAIRE | ORTSTOM | Marine | atlas | Missing or invalid geospatial data | NO | YES | Atoll, island or archipelago | personal_copy | NA |
| 1992 | Nukutipipi Atoll, Tuamotu Archipelago; Geomorphology, land and marine flora and fauna and interrelationships | Salvat F., Salvat R. | Marine & Terrestrial | article | Missing or invalid taxonomical data | NO | NO | None | <https://repository.si.edu/bitstream/handle/10088/5035/00357.pdf> | No dataset and no precise coordinates |
| 1990 | Spatial pattern in the abundance and structure of mollusc populations in the soft sediments of a coral reef lagoon | Jones G., et al. | Marine | article | Unpublished or unavailable data | NO | YES | Field ID | <https://www.jstor.org/stable/pdf/24842499.pdf?refreqid=excelsior%3Adf0cf91d60913e3dd9f17098ca31b019&ab_segments=&origin=&initiator=&acceptTC=1> | Unpublished |
| 1922 | International ornithological biological data | Birdlife | All | website | Unpublished or unavailable data | NO | NA | NA | <http://datazone.birdlife.org/home> | Spatial data requires a request form submission |
